# An Alternative Phosphorylation Switch in Integrin β2 (CD18) Tail for Dok1 Binding

**DOI:** 10.1038/srep11630

**Published:** 2015-06-25

**Authors:** Sebanti Gupta, Joel Chia-Yeong Chit, Chen Feng, Anirban Bhunia, Suet-Mien Tan, Surajit Bhattacharjya

**Affiliations:** 1 School of Biological Sciences, Nanyang Technological University, 60 Nanyang Drive, Singapore 637551.

## Abstract

Integrins are involved in cell migration and adhesion. A large number of proteins interact with the cytoplasmic tails of integrins. Dok1 is a negative regulator of integrin activation and it binds to the phosphorylated membrane proximal NxxY motif in a number of integrin β tails. The β tail of the β2 integrins contains a non-phosphorylatable NxxF motif. Hence it is unclear how Dok1 associates with the β2 integrins. We showed in this study using NMR and cell based analyses that residues Ser745 and Ser756 in the integrin β2 tail, which are adjacent to the NxxF motif, are required for Dok1 interaction. NMR analyses detected significant chemical shift changes and higher affinity interactions between Dok1 phospho-tyrosine binding (PTB) domain and integrin β2 tail peptide containing pSer756 compared to pSer745. The phosphorylated β2 peptide occupies the canonical ligand binding pocket of Dok1 based on the docked structure of the β2 tail-Dok1 PTB complex. Taken together, our data suggest an alternate phosphorylation switch in β2 integrins that regulates Dok1 binding. This could be important for cells of the immune system and their functions.

Integrins are a large family of cell surface αβ heterodimers that mediate cell-cell and cell-ECM interactions necessary for many physiological processes, including hemostasis, wound healing, immunity and developmental biology^1^. Each subunit of an integrin has a large extracellular region that binds to ligands, a single-pass transmembrane domain that transduces activation signal across the plasma membrane and a short cytoplasmic tail (except integrin β4) that binds to an expanding list of cytoplasmic proteins^2^. Except β4 and β8, integrin β tails contain two highly conserved NxxY/F (x: other amino acid) motifs that are docking sites for cytoplasmic proteins^2^. The membrane proximal NxxY/F motif is a binding site for talin, a well-established cytoskeletal protein that directly activates integrins^3^,^4^. The two isoforms of talin in vetebrates (talin 1 and 2) are 4.1-ezrin-radixin-moesin (FERM)-containing proteins^5^,^6^. The FERM domain lies in the head region of talin, and a phosphotyrosine binding (PTB) fold in its F3 subdomain has been shown to bind the membrane proximal NPLY747 motif of the integrin β3 tail^7^. This form of interaction is not limited to talin because it extends to other cytoplasmic proteins containing PTB folds, including negative regulator of Notch signaling (Numb), downstream target of c-Abl (Dab) and docking protein 1 (Dok1; p62Dok)^8^.

Dok1 is a member of the Dok family of adaptor proteins and it is expressed in lymphoid and myeloid cells^9^,^10^. Dok1 and Dok2 are negative regulators of immune cell signaling and Dok1 has been reported to bind with p120RasGAP, a negative regulator of the Ras-ERK pathway^11^. All seven members of the Dok family of proteins contain an N-terminal pleckstrin homology (PH) domain, a central PTB fold and multiple SH2 and SH3 binding sites^11^. Dok1 and talin have overlapping binding sites which include the NPLY747 motif in the integrin β3 tail. Therefore it is unlikely that both molecules can simultaneously bind the integrin β tail. Indeed biophysical analyses have shown that phosphorylated Y747 enhances Dok1 binding over that of talin, suggesting that NPLY747 is a phosphorylation switch^12^. Unlike talin, Dok1 does not activate integrins. Dok1 is a negative regulator of integrin activation by competing with talin for binding to integrin β1A, β3 and β7 tails containing the membrane proximal NxxY motif^13^,^14^. The leukocyte-restricted β2 integrins comprise four members that have different α subunits but a common β2 (CD18) subunit, namely αLβ2, αMβ2, αXβ2 and αDβ2^2^. The importance of the β2 integrins is underscored by the rare autosomal disease Leukocyte Adhesion Deficiency (LAD) I in which afflicted individuals have a compromised immune system because of defective adhesive and migratory properties of their leukocytes. The molecular basis of LAD I is the reduced expression and/or expression of dysfunctional β2 integrins in leukocytes as a result of mutation(s) in the *ITGB2* gene^15^,^16^. Dok1 has been reported to bind the integrin β2 tail^8^. However, the corresponding Dok1 binding region in β2 contains an NPLF754 motif that does not allow phosphorylation. This begs the question if there is an alternative phosphorylation site(s) in the region that regulates Dok1 binding. Residues Ser745 and Ser756 flank the NPLF754 motif in the integrin β2 tail. The corresponding Ser residues are absent from the β tails of β3, β5 and β6 integrins (Table 1). However, the β tails of β1A, β1D and β7 integrins contain a Ser residue at an equivalent position to that of Ser 756 in the β2 tail. *In vitro* studies have shown that integrin β2 Ser745 and Ser756 are phosphorylatable but only the former is dependent on PKCs in T cells^17^. These observations suggest an interesting possibility that the phosphorylation state of Ser745 and Ser756 could regulate the binding of Dok1 to integrin β2 tail.

In this study, we showed using NMR that the integrin β2 tail interacts with the PTB domain of Dok1. Phosphorylation at Ser756 of the integrin β2 tail enhanced its affinity towards the PTB domain of Dok1. Similar observations were made using a 15-residue peptide fragment containing phosphorylated Ser756. ^15^N-^1^H HSQC spectra, ^31^P NMR, tr-NOESY, saturation transfer difference (STD) NMR and HADDOCK docking provided insights for the first time into the mode of binding and conformations of the integrin β2 tail in complex with Dok1 PTB domain. The importance of Ser745 and Ser756 in the integrin β2 tail for Dok1 binding was verified by cell based FRET analysis. In addition, we showed that cellular activation by chemokine induced the dissociation of Dok1 from the integrin β2 tail. The current findings therefore suggest a novel paradigm involving an alternative phosphorylation switch in the integrin β2 tail that is required for the regulation of β2 integrins by Dok1.

## Results

### Binding of the PTB domain of Dok1 with phosphorylated full-length integrin β2 tail peptides

**Figure 1:**
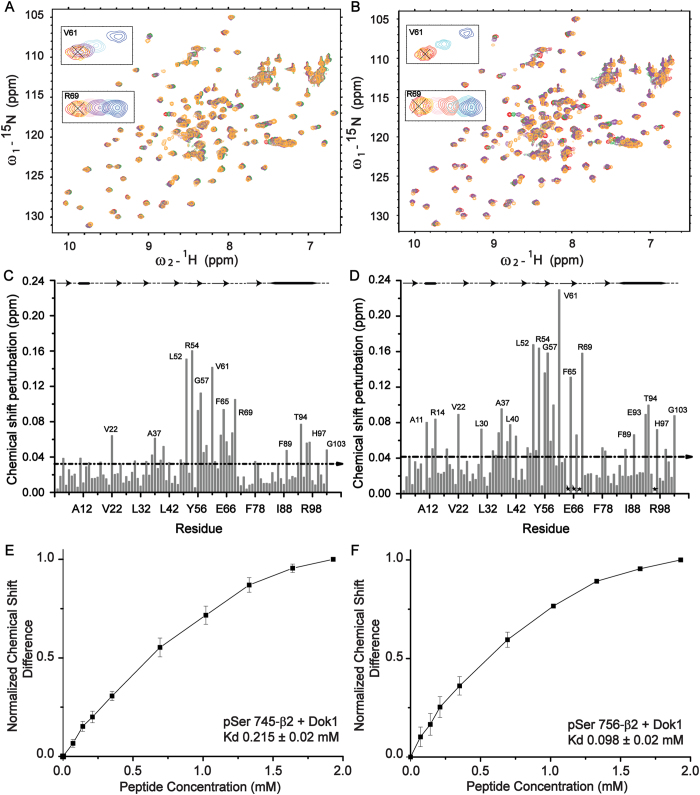
Dok1 binds to the integrin β2 tail upon phosphorylation of Ser745 and Ser756. (**A** and **B**) represent the ^15^N-^1^H HSQC spectra of the Dok1 PTB domain, showing chemical shift changes at 0 mM (red contour), 0.4 mM (cyan contour), 1 mM (violet contour) and 2 mM (orange contour) concentrations of pSer745 (**A**) and pSer756 β2 tail (**B**). (in inset) Chemical shift changes of residue Arg69 and Val61 of the PTB domain of Dok1 are shown. Bar diagrams showing combined chemical shift perturbation for ^15^N and HN resonances of each residue of the Dok1 PTB domain upon binding to pSer745-β2 (**C**) and pSer756-β2 (**D**). Note in (**D**) residues showing resonance broadening upon additions of phosphorylated β2 tail are marked as asterisks. The dotted lines in (**C** and **D**) marked average chemical shift perturbation. The secondary structural elements are shown at the top of each plot. Normalized chemical shift differences are plotted against the concentrations of pSer745-β2 (**E**) and pSer756-β2 (**F**) for Dok1 PTB domain to determine equilibrium dissociation constants (K_d_) values.

**Figure 2:**
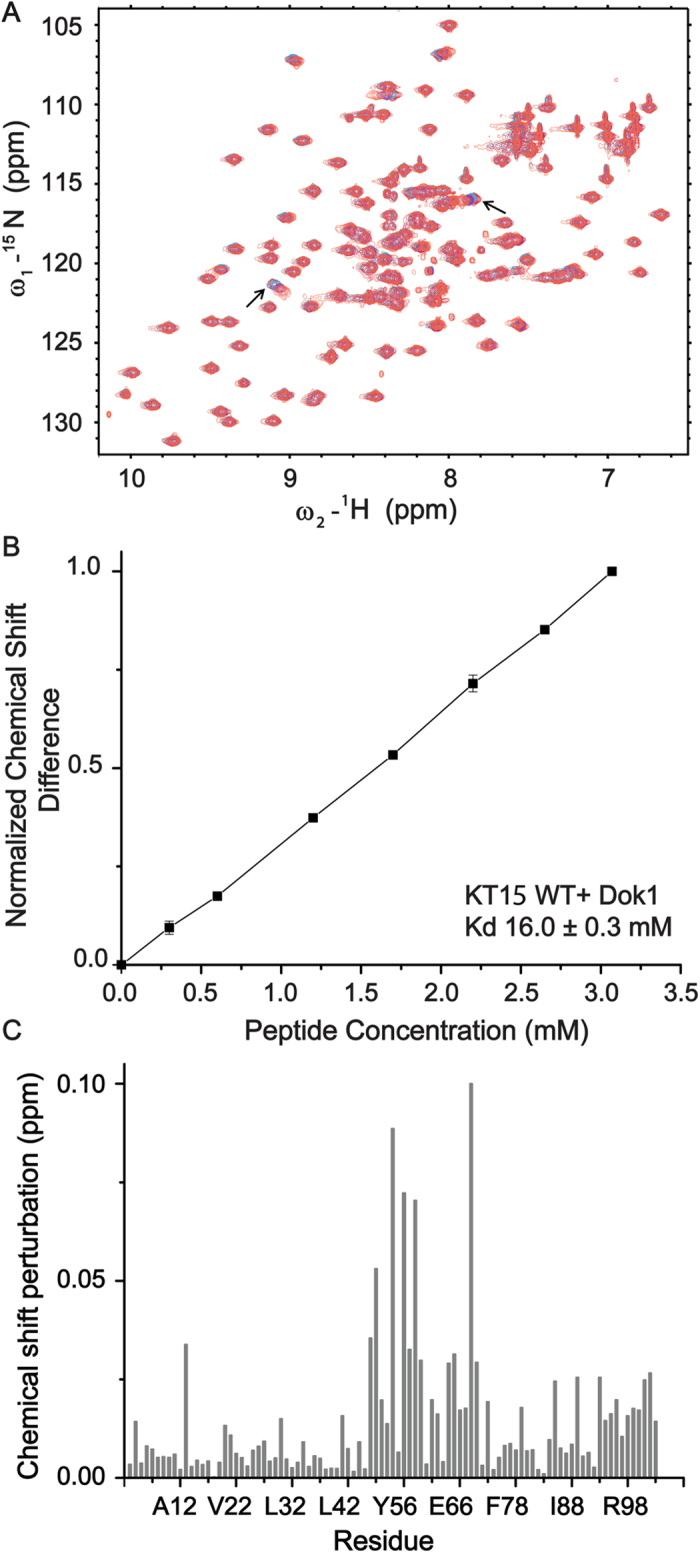
Interactions of Dok1 with unphosphorylated β2 tail KT15 peptide. (**A**) Overlay of ^15^N-^1^H HSQC spectra of the Dok1 PTB domain, showing chemical shift changes at protein:peptide concentration ratios 1:0 (red contour), 1:1 (cyan contour), 1:3 (violet contour) and 1:6 (orange contour). Two HSQC peaks corresponding to residue Arg54 at ~9.00 ppm and residue Tyr56 at ~7.9 ppm showed KT15 binding induced perturbation (arrows). (**B**) Normalized chemical shift differences for Dok1 PTB domain are plotted against the concentrations of KT15 peptide to determine the K_d_ value. (**C**) Bar diagram showing combined chemical shift perturbations for ^15^N and HN resonances of each residue of the Dok1 PTB domain upon binding to KT15 peptide (protein:peptide concentration ratio 1:6).

**Figure 3:**
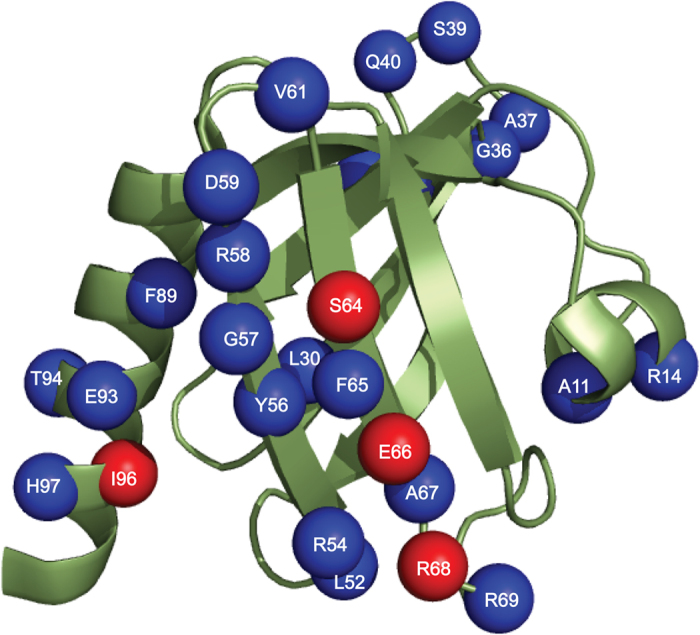
Mapping residues of Dok1 PTB domain that interact with pSer756-β2 tail. A ribbon representation of the three-dimensional structure of the Dok1 PTB domain (pdb: 2v76). Residues that exhibited above average chemical shift perturbations and resonance broadening in the presence of pSer756-β2 tail are shown as blue spheres and red spheres, respectively.

Interactions between Dok1 PTB domain with full-length integrin β2 tail phosphorylated either at Ser745 (pSer745-β2) or Ser756 (pSer756-β2) were investigated by analyzing the changes in ^15^N-^1^H HSQC spectra of the PTB domain. In these experiments, a series of ^15^N-^1^H HSQC spectra of ^15^N-labelled PTB domain were acquired at various concentrations of integrin β2 tail peptides (Fig. 1A,B). ^15^N-^1^H HSQC spectra of the PTB domain showed discernable chemical shift changes upon addition of phosphorylated integrin β2 tail peptides. The chemical shifts of the backbone resonances including ^15^N and ^1^H of the Dok1 PTB domain have been previously assigned^12^,^18^. Chemical shift perturbations of a number of residues including Leu52, Arg54, Tyr56, Gly57, Val61, Phe65, Arg69 at the central region of the Dok1 PTB domain were detected when pSer745-β2 or pSer756-β2 peptide was added (Fig. 1C,D). However, the chemical shift changes were more pronounced in the presence of pSer756-β2. Residues Ala11, Arg14, Val22, Leu30, Ala37, Gln40 at the N-terminus and residues Phe89, Glu93, Thr94, His97 at the C-terminus of Dok1 also showed chemical shift perturbations (Fig. 1D). The binding affinity of Dok1 PTB domain with phosphorylated integrin β2 tail was determined from the combined chemical shift changes in the presence of increasing ligand concentrations with K_d_ values of 98 μM and 215 μM for pSer756-β2 and pSer745-β2 tail peptides, respectively (Fig. 1E,F). These data suggest that phosphorylation at Ser756 as compared to Ser745 of the integrin β2 tail promotes higher affinity interaction with Dok1 PTB (Table 2). Due to limited solubility of the full-length unphosphorylated β2 tail at high concentrations, ^15^N-^1^H HSQC spectra of Dok1 PTB domain were obtained using a fragment of the integrin β2 tail KSQWNNDNPLFKSAT or KT15 that contains both serines. Few chemical shift changes of residues in Dok1 PTB domain in the presence of unphosphorylated KT15 were detected (Fig. 2A). The extent of chemical shift changes of Dok1 in the presence of KT15 was also limited (Fig. 2B). An apparent K_d_ value of 16 mM was determined for the interaction between Dok1 PTB domain and unphosphorylated **KT**15 (Fig. 2C, Table 2). Compared with pSer756-β2 and pSer745-β2 tails, the unphosphorylated integrin β2 tail interacts weakly with the Dok1 PTB domain. Similar observations were made in interactions studies using unphosphorylated integrin β3 tail with the Dok1 PTB domain^12^. Chemical shift perturbations caused by pSer756-β2 tail were mapped onto the 3-D structure of Dok1 PTB domain (Fig. 3). Most of the binding residues are located in the C-terminal α-helix and the β5 strand-loop-β6 strand-loop of the seven stranded β-sheet of the PTB domain (Fig. 3). These structural elements of Dok1 PTB domain are involved in binding pTyr peptide ligands^12^,^19^.

### Conformations and interactions of phosphorylated integrin β2 tail fragment with PTB domain

**Figure 4:**
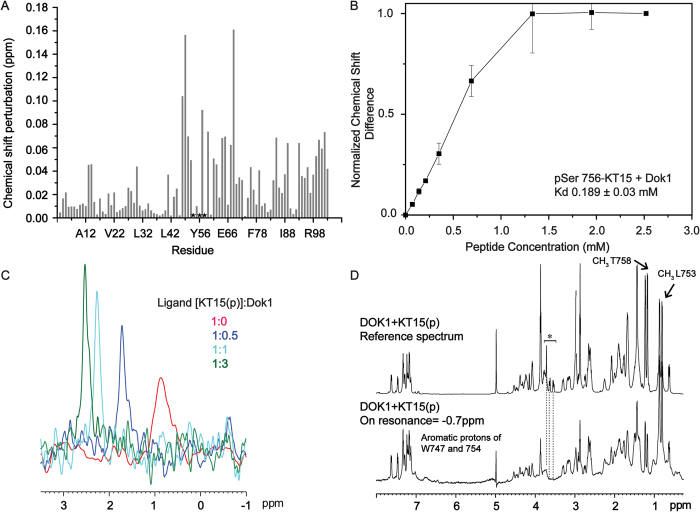
Analyses of interactions of pSer756 KT15 peptide with Dok1 PTB domain. (**A**) Bar diagram showing combined chemical shift perturbations for ^15^N and HN resonances of residues of the Dok1 PTB domain upon binding to pSer756 KT15 peptide. Residues showing resonance broadening are marked (asterisks). (**B**) Normalized chemical shift differences for Dok1 PTB domain are plotted against the concentrations of pSer756 KT15 peptide to determine the K_d_ value. (**C**) ^31^P NMR spectra of pSer756 KT15 peptide in free solution and in the presence of Dok1 PTB domain at different peptide:protein molar ratios (1:0 (red), 1:0.5 (blue), 1:1 (cyan) and 1:3 (green)). (**D**) Saturation transfer difference (STD) NMR spectrum of pSer756-KT15 peptide in the presence of Dok1 PTB domain. The off-resonance or the reference spectrum is shown (top).

To gain further insight into phosphorylated integrin β2 tail-Dok1 interactions, we made use of a pSer756-KT15 (KSQWNNDNPLFKpSAT) peptide. ^15^N-^1^H HSQC spectra of the Dok1 PTB domain were acquired at various concentrations of pSer756-KT15 peptide. Chemical shift perturbations were detected for residues of Dok1 PTB domain located at the canonical ligand binding site akin to that detected using the full-length pSer756-β2 tail (Fig. 4A). A K_d_ value of 189 μM was determined for the interaction between pSer756-KT15 the and Dok1 PTB domain (Fig. 4B, Table 2). This value is somewhat higher than the K_d_ value of 98 μM obtained using full-length pSer756-β2 tail peptide. This may suggest additional interactions between full-length integrin β2 tail with the Dok1 PTB. To probe the direct involvement of the phosphate group on Ser756, we acquired ^31^P NMR spectra of pSer756-KT15 peptide in aqueous solutions in the absence or presence of different concentrations of Dok1 PTB domain (Fig. 4C). The ^31^P NMR signal of pSer756-KT15 peptide resonated at ~0.9 ppm in free solution. There was a significant downfield shift (toward higher chemical shift values) of the ^31^P NMR signal upon addition of Dok1 PTB domain. ^31^P NMR resonance of pSer756 shifted to as much as ~2.5 ppm at the protein to peptide ratio of 3:1. This large downfield shift or deshielding effect of ^31^P resonance of pSer756-KT15 peptide could be attributed to ionic and/or hydrogen bond interactions with the residues of Dok1 PTB domain.

We performed isotope filtered 3-D NOESY experiments with ^15^N/^13^C labelled samples of Dok1 PTB domain with unlabeled pSer756-KT15 peptide to determine the atomic-resolution structure of the PTB domain-β2 peptide complex. However, it was unsuccessful because of the paucity of inter-molecular NOEs, possibly due to fast dissociation of the complex and relatively insensitive nature of filtered experiments (data not shown). tr-NOESY^20^,^21^ and STD-NMR^22^,^23^ methods were used to determine the bound structure and interactions of pSer756-KT15 peptide ligand and Dok1 PTB domain. Others have reported that a fast chemical exchange between the free-state and bound-state of ligands are necessary for tr-NOEs and STD experiments^20^,^21^,^22^,^23^. ^15^N-^1^H HSQC spectra of Dok1 PTB domain with pSer756-KT15 peptide indicated fast exchange regime of complex formation. tr-NOESY spectra of pSer756-KT15 peptide showed only sequential and intra-residue NOEs, suggesting that the pSer756-KT15 peptide adopts an extended or a β-strand-like conformation (Supplementary Fig. 1). 1-D STD spectrum and the rence or off-resonance spectrum of pSer756-KT15 peptide in the presence of Dok1 PTB domain were analyzed (Fig. 4D). STD spectrum of pSer756-KT15 peptide appears to retain several signals and is largely similar to the reference spectrum, indicating intimate association of pSer756-KT15 peptide with the PTB domain. A detailed group epitope mapping analyses of the pSer756-KT15 peptide in complex with Dok1 is impeded due to overlapping resonances in the 1-D STD spectrum. However, STD effects arising from diagnostic resonances can be ascertained. In particular, downfield shifted aromatic resonances at 7.2 to 7.7 ppm observed in the STD spectrum reveal proximity of the aromatic ring of residues Trp747 and Phe754 of pSer756-KT15 peptide to the Dok1 PTB domain. The upfield resonances at 0.8–0.9 ppm and 1.25 ppm of the alkyl sidechain of single Leu753 and CH_3_ group of single Thr758 can be seen in the STD spectrum, indicating their proximity with the PTB domain. pSer756-KT15 peptide did not exhibit any discernible STD effect in free solution (Supplementary Fig. 2). STD experiments were also performed with unphosphorylated KT15 peptide with Dok1 PTB domain but there was apparently no or low STD effect, indicating a lack of binding with the Dok1 PTB domain (Supplementary Fig. 3).

### Docking of pSer756-KT15 peptide with Dok1 PTB domain

**Figure 5:**
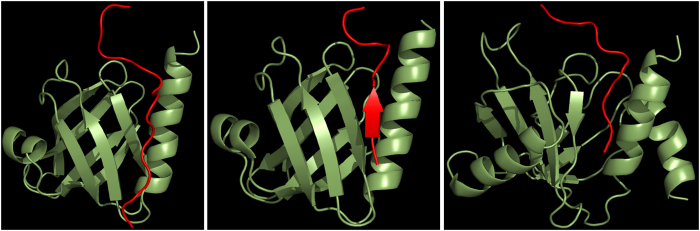
Binding mode and orientation of pSer756 KT15 peptide in complex with Dok1 PTB domain. (left panel) A ribbon representation of the overall topology of the Dok1 PTB domain (green) in complex with pSer756-KT15 peptide (red) obtained from docking simulation. (middle panel) X-ray structure of Dok1 PTB domain (green) in complex with Tyr phosphorylated RET peptide (red). (right panel) NMR structure of the Numb PTB domain (green) in complex with Nak peptide (red).

**Figure 6:**
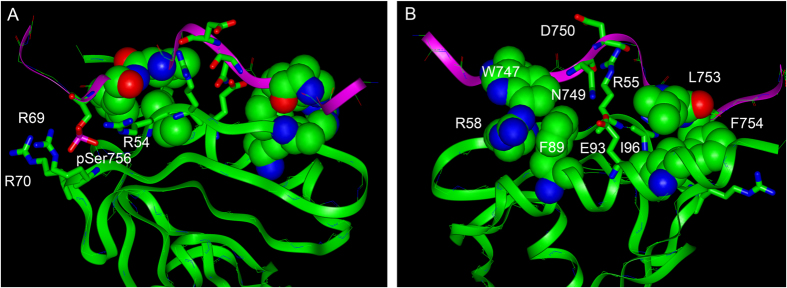
Molecular interactions of pSer756 KT15 peptide with Dok1 PTB domain. (**A**) The docked model of pSer756-KT15/Dok1 PTB domain suggests potential salt bridge/hydrogen bond interactions between the phosphate group of pSer756 and the side chain guanidinium groups of Arg54, Arg69 and Arg70 of Dok1. (**B**) The probable aromatic-aromatic stacking and cation-π interactions of Dok1 PTB domain Phe89 and Arg58 with Trp747 of pSer756-KT15 peptide are shown. Hydrophobic packing interactions between Phe754 and Leu753 in the NPLF motif of pSer756-KT15 peptide with Ile96 of the PTB domain are observed. In addition, there can be polar and ionic interactions: Asp749 (K15) with Glu93 (Dok1) and Asp750 (K15) with Arg55 (Dok1).

We utilized the structural information of Nak peptide in complex with the PTB domain of Numb protein for modeling the backbone conformation of the pSer756-KT15 peptide (See Materials and Methods)^24^. The 11-residue Nak peptide adopts largely extended conformation when in complex with Numb PTB domain^24^. The sequence of Nak peptide, GFSNMSFEDFP, shows some similarity with pSer756-KT15 peptide in terms of the composition of residues and the residues that are involved in binding to the target protein. Two Phe in the Nak peptide, GFxxxxxxxFP, define a sequence pattern similar to WxxxxxxF in the pSer756-KT15 peptide. Overall topologies of Dok1 PTB domain in complex with pSer756-KT15 (left panel), RET peptide (middle panel) and Numb PTB domain with the Nak peptide (right panel) are shown (Fig. 5). In these structures, PTB domain-peptide complexes are defined by extended conformations of the peptide ligands occupying the binding region comprising the C-terminal long helix and β5 and β6 strands. The peptides are also in close association with the surface of the PTB domains. In the docked structure of pSer756-KT15/Dok1 PTB domain, there are a number of potential molecular interactions that may stabilize the complex. The phosphate group of pSer756 is in close proximity to the side-chain guanidinium groups of residues Arg54, Arg69 and Arg70 of the PTB domain, suggesting salt-bridge and/or hydrogen bond interactions (Fig. 6A). These Arg residues are also involved in the recognition of the pTyr in RET and integrin β3 tail peptide^12^,^19^. Apart from these interactions, aromatic-aromatic stacking and cation-π contacts may be involved between Dok1 PTB domain Phe89 and Arg58 with Trp747 of the pSer756-KT15 peptide (Fig. 6B). Hydrophobic packing interactions may occur between residues Phe754 and Leu753 in the NPLF motif of pSer756-KT15 peptide with residue Ile96 of the Dok1 PTB domain. In addition, polar and ionic interactions appear to be sustained between residues Asn749/Glu93, Asp750/Arg55 of pSer756-KT15 peptide and Dok1 PTB domain (Fig. 6B).

### Cell-based analyses of Dok1 binding to integrin β2 cytoplasmic tail

**Figure 7:**
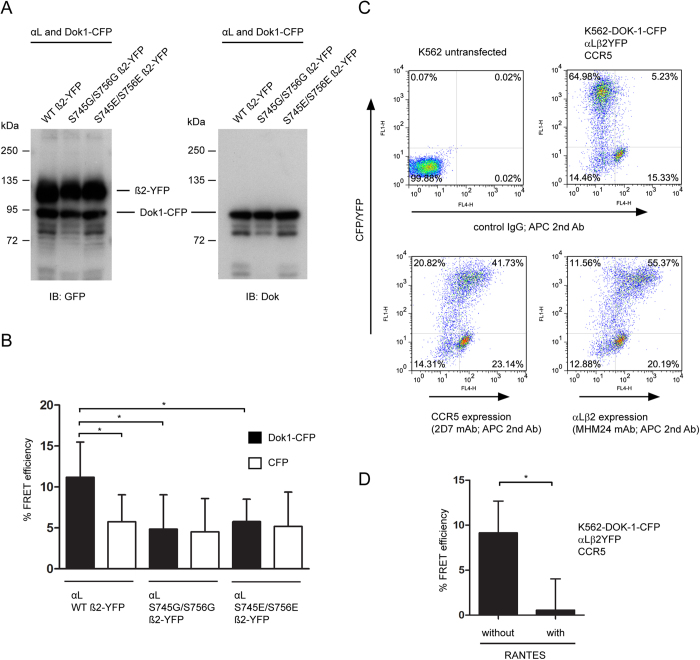
Analyses of the interaction between integrin β2 tail and Dok1 in cells. (**A**) K562 cells transfected with the indicated expression plasmids were lysed. Proteins were resolved on 10% SDS-PAGE under reducing conditions and immunoblotted (IB) with either anti-GFP or anti-Dok1 antibodies. (**B**) Transfected K562 cells were subjected to YFP-photobleach FRET analyses. Each data point represents the mean ± S.D. of ≥30 cells analyzed. (**C**) Flow cytometry analyses of K562 stable line expressing Dok1-CFP that were transfected with integrin αLβ2-YFP and CCR5. Wild-type K562 cells were used as the control group. (**D**) FRET analyses of cells in (**C**) that were treated without or with chemokine RANTES (50 ng/ml) for 10 min at 37 °C. 60 and 39 cells were analyzed for conditions without and with RANTES treatment, respectively. Data point represent mean ± S.D. *,p < 0.05, Student’s *t* test.

We examined the binding of Dok1 to integrin β2 cytoplasmic tail in cells using FRET assay. The myeloid leukemia K562 cells that do not express endogenous β2 integrins were transfected with expression plasmids of integrin αL and Dok1-CFP (CFP fused to the C-terminus of Dok-1) and integrin β2-YFP wild-type or S745G/S756G or S745E/S756E. Since the integrin β2 tail assumes extended conformation in complex with Dok1, we have chosen Ser to Gly mutations in the β2 tail. The technical control groups were cells transfected with the indicated plasmids but the Dok1-CFP was substituted with empty CFP plasmid. The expression levels of integrin β2-YFP constructs and Dok1-CFP were examined by immunoblotting (Fig. 7A). Transfected cells were subjected to YFP-photobleach FRET analyses. Significant FRET signal was detected in cells transfected with αL, Dok1-CFP and integrin β2-YFP wild-type compared with control group cells transfected with αL, CFP and integrin β2-YFP. Cells transfected with either integrin β2 S745G/S756G or S745E/S756E had significantly reduced FRET signal. These data suggest that Dok1 binds to the integrin β2 cytoplasmic tail and the association requires β2 Ser745 and/or Ser756. However, poor FRET signal was detected when integrin β2 Ser745 and Ser756 were both substituted with the acidic residue Glu to mimick phosphorylation. This is line with our NMR experiments that showed weak interactions (K_d_ ~ 8.49 mM) between β2 peptide fragment containing Ser to Glu substitutions with the Dok1 PTB domain (Table 2, Supplementary Fig. 4). Ser to Glu or Asp substitution has been commonly used to interrogate the properties of phosphorylated Ser but the inability of Asp/Glu to mimic phosphorylated Ser has also been reported^25^. Hence, it is possible that the single carboxylate group on the side chain of Glu is insufficient to reconstitute the double negative charge and bulky phosphate moiety on phosphorylated Ser. We have attempted to detect the phosphorylation of integrin β2 cytoplasmic tail on Ser745 and Ser756 by immunoprecipitation of wild-type integrin αLβ2 from transfected K562 cells and the T cell line Jurkat followed by immunoblotting with either an anti-phospho Ser antibody or anti-integrin β2 phospho Ser745 obtained from commercial sources. However, we were unable to obtain definitive results possibly due to poor specificity and reactivity of these antibodies (data not shown).

Dok-1 binds integrin β cytoplasmic tail and it is a negative regulator of integrin activation^13^,^14^. To date, however, there is a lack of cell-based evidence demonstrating Dok-1 dissociating from integrin β cytoplasmic tail in the presence of an activation signal. Hence we examined if chemokine-induced cell activation leads to the dissociation of Dok-1 from the integrin β2 cytoplasmic tail. The chemokine RANTES and its receptor CCR5 were used in our study because RANTES has been reported to stimulate leukocyte adhesion and migration via activation of integrin αLβ2^26^,^27^. We generated stable K562 expressing Dok1-CFP by antibiotic selection. These cells were then transfected with expression plasmids of integrin αL and β2-YFP and CCR5. The expression levels of these proteins were verified by flow cytometry analyses (Fig. 7C). Cells were then treated without or with RANTES and FRET analyses performed on these cells (Fig. 7D). FRET signal was significantly lower in cells treated with RANTES compared with untreated cells. These data suggest that Dok-1 dissociates from integrin β2 cytoplasmic tail in the presence of RANTES/CCR5 activation signal.

## Discussion

A large number of cytosolic proteins, including talins, kindlins, filamins, Dok1, 14-3-3, are known to interact with the β tail of integrins and they regulate the ligand-binding activity of these integrins^28^,^29^,^30^. Interactions with cognate proteins can be modulated by phosphorylation of the integrin β tail at specific sequence motifs^12^,^13^,^28^. The membrane proximal NxxY motif found in the β tail of a number of integrins (Table 1) interacts with the talin head domain, IgFLN domains of filamin and PTB domain of Dok1^2^,^28^,^29^,^30^. Phosphorylation of Tyr in the NPxY motif enables binding of the Dok1 PTB domain which negatively regulate the activation of integrins^12^,^13^,^31^. The PTB domain of Dok1 has also been shown to bind to a Tyr phosphorylated synthetic peptide derived from the RET receptor tyrosine kinase^19^. The membrane proximal NPxF motif in the integrin β2 tail is not a phosphorylation site. Others have reported that Ser745 and Ser756, which are adjacent to the membrane proximal NPxF in the integrin β2 tail, are amenable to phosphorylation^17^. This led us to hypothesize that an alternate phosphorylation switch involving Ser745 and Ser756 regulates the interaction of Dok1 PTB with the integrin β2 tail. Previous studies have demonstrated the functional consequences of Ser745 and Ser756 phosphorylation in integrin β2 tail^32^,^33^,^34^. However, there has been no in depth study to our knowledge demonstrating an integrin β2 tail binding protein that recognizes these phosphorylated serines. This study demonstrates that the PTB domain of Dok1 binds to integrin β2 tail, which is dependent on the phosphorylation state of Ser745 and Ser756. NMR experiments using phosphorylated full-length integrin β2 tail peptides showed that the affinity of Dok1 PTB was higher with integrin β2 tail having phosphorylated Ser756 compared with phosphorylated Ser745. It is noteworthy that the integrin β1A, β1D and β7 tails also contain a potential phosphorylatable Ser residue at the equivalent position of Ser756 in the β2 tail (Table 1). pSer756-β2 tail occupies the canonical binding pocket of Tyr phosphorylated peptides at the C-terminal long helix and β5-β6 strands of the PTB domain. The docked structure of Dok1 PTB with the integrin β2 pSer756-KT15 peptide provides information on their relative orientation and the molecular interactions involved. In this model, the phosphate group of pSer756-KT15 is found in a close proximity to the side chains of residues Arg54 and Arg69 of the PTB domain that allows the formation of ionic interactions and/or hydrogen bonds. Indeed ^31^P NMR spectra of pSer756-KT15 peptide showed significant chemical shift changes when in complex with the Dok1 PTB domain (Fig. 4C) and ^15^N-^1^H HSQC spectra of the PTB domain showed high chemical shift perturbations of these corresponding Arg residues upon addition of the phosphorylated integrin β2 tail peptide (Fig. 1D). The docked structure of the phosphorylated integrin β2 peptide/PTB domain suggests packing interactions between residues Leu731 and Phe732 in the NPLF motif and Trp725 of the β2 tail with residue Ile96 and residues Arg58 and Phe89 of the PTB domain, respectively (Fig. 6). STD-NMR studies demonstrated that the aromatic ring of residues Trp725 and Phe732, and the aliphatic side chains of residues Leu731 and Thr758 are in close proximity to the PTB domain of Dok1 (Fig. 4D). Alongside, Ile96, Arg58 and Phe89 of the PTB domain demarcated chemical shift perturbations when in complex with the phosphorylated β2 tail (Fig. 1D). The binding affinity of the phosphorylated integrin β2 tail with the PTB domain of Dok1 is rather low. We hypothesize that in a physiological system, this binding affinity can be enhanced by their membrane localization. Indeed, the PH domain of Dok1 protein has been shown to be important for Dok1 targeting to the membrane^35^. Interactions between integrin tails and their binding partners are found to be of higher affinity when the integrin tails are anchored in lipids^36^,^37^. It has also been reported that membrane interactions stabilize the binding of the head domain of talin to integrin tails^38^,^39^. Although we have not examined the interaction of di-phosphorylated (pSer745 and pSer756) integrin β2 tail peptide with Dok1 PTB domain in this study, we conjecture that the binding affinity may be further enhanced when both Ser are phosphorylated. This will be investigated in future work. In a cellular system, the simultaneous phosphorylation of both Ser745 and Ser756 could have a synergistic effect on the recruitment of Dok1 to the integrin β2 tail. In this regard, phosphorylation of Ser745 or Ser756 or both under different conditions may allow fine-tune regulation of Dok1 binding to integrin β2 tail, which may in turn modulate talin activation of β2 integrins. In addition to biophysical analyses, we showed using a cell-based FRET system that the interaction between integrin β2 tail and Dok1 is dependent on Ser745 and Ser756, and cellular activation by chemokine stimulation triggers the dissociation of Dok1 from the integrin β2 tail. We were unable to demonstrate the phosphorylation status of integrin β2 Ser745 and Ser756 in cells because antibodies of high specificity and reactivity are unavailable to us. However, others have shown that these residues can be phosphorylated^17^,^34^.

An increasing number of cytosolic proteins have been identified to bind integrin tails. These interactions are regulated by many factors including the phosphorylation of integrin β tails. Given that there are sequence variations amongst the integrin β tails, there can be slight but important differences in which phosphorylation regulates these interactions. In this study, we have identified and characterized an alternate phosphorylation switch in the integrin β2 tail that regulates its interaction with Dok1. These findings will be important for future studies investigating the underlying mechanisms of β2 integrin-mediated immune responses.

## Materials and Methods

### Plasmids

Full-length Dok1 cDNA from a human leukocyte cDNA library (Clontech Laboratories, Mountain View, CA) was PCR amplified using relevant primers and cloned into the plasmid pcDNA3.1(+) (Invitrogen, Carlsbad, CA). Dok1 was PCR sub-cloned into the pECFP-N1 expression plasmid (Clontech) for FRET experiments. The pcDNA3 expression plasmids containing human integrin αL or integrin β2-YFP (YFP fused to the C-terminal of β2 cytoplasmic tail) have been reported previously^40^,^41^,^42^. The integrin β2-YFP S745G/S756G expression construct was generated by mutagenesis using the Quikchange II site-directed mutagenesis kit (Stratagene, La Jolla, CA) and relevant primers. The human CCR5 pcDNA3 expression plasmid was a kind gift from Dr. R.W. Doms (MD) (University of Pennsylvania, PA).

### Cell culture, transfection and fluorescence resonance energy transfer (FRET) experiments

K562 cells (ATCC, Manassas, VA) were cultured in RPMI1640 full-medium containing 10% (v/v) FBS and antibiotics. Cells (2 × 10^6^) were transfected with integrin αL plasmid (9 μg), integrin β2-YFP plasmid (WT or mutant) (9 μg) and Dok1-CFP or CFP plasmid (12 μg) by electroporation using the Neon electroporation kit (Invitrogen) on a MP-100 pipette-type microporator (Invitrogen). YFP-photobleach FRET assay was performed essentially as described^40^. Immunoblottings of transfected K562 lysates were performed using rabbit anti-GFP antibody (Life Technologies, Grand Island, NY), rabbit anti-Dok1 antibody (Abcam, Cambridge, MA) and HRP-conjugated goat anti-rabbit (Avansta, Menlo Park, CA) followed by ECL detection. To generate K562 cells stably expressing Dok1-CFP, cells were transfected with Dok1-CFP plasmid by electroporation followed by serial dilutions into 96-well cell culture microtitre plates to select for stable clones in the presence of antibiotic G418 (0.6 mg/ml). Clones expressing Dok1-CFP were screened by flow cytometry analyses and verified by immunoblotting with anti-GFP antibody (Life Technologies, Grand Island, NY). Stable K562 cells expressing Dok1-CFP transfected with integrin αL, integrin β2-YFP and CCR5 plasmids (12 μg each) were stained with either 1 μg each of mAb MHM24 (anti-αL)^41^ or mAb 2D7 (anti-CCR5, BD Biosciences, San Jose, CA) followed by APC-conjugated secondary antibody (goat anti-mouse IgG, BD Biosciences, 1:500 dilution). Samples were acquired and examined on a FACSCalibur flow cytometer and data analyzed using the Flowjo software (Tree Star Inc. Ashland, OR). For RANTES activation, transfected K562 cells were incubated in culture medium containing RANTES (50 ng/ml) (Calbiochem, Merk Millipore) for 10 min at 37 °C before performing FRET analyses.

### Protein expression and purification

Human Dok1 PTB domain (Q154 – G256; Swiss-Prot Q99704, number as Q1-G103) was sub-cloned into the pET14b vector with an N-terminal six His-tag. *Escherichia coli* Rosetta cells were transformed with the expression plasmid and grown at 37 °C either in LB or M9 medium containing [^15^N] ammonium chloride (Cambridge Isotope Laboratories). Expression of recombinant protein was induced by adding 1 mM IPTG to cells (OD_600_ 0.6–0.7) followed by incubation for 6–12 h at 16 °C. Cells were centrifuged and re-suspended in Buffer A (20 mM Tris-HCl buffer, pH 8.0) followed by affinity purification on a Nickel-NTA column (Qiagen). His-tagged Dok1 PTB domain was eluted in Buffer A containing 500 mM imidazole and dialyzed against Buffer B (50 mM sodium phosphate buffer (pH 6.0) containing 100 mM NaCl and 2 mM DTT). Protein samples were further purified by gel filtration in Buffer B on a Hiload Superdex 75 16/26 GL preparative column that was connected to an AKTA FPLC UPC-900 system (GE Healthcare UK Ltd., England). Proteins were eluted at a flow rate of 0.5 ml/min and monitored by absorbance at λ = 276 nm.

### Synthetic peptides

Synthetic peptides (purity >95%) of full-length β2 tail, phosphorylated full-length β2 (pSer745-β2 and pSer756β2), phosphorylated peptide fragment of β2 (pSer756-KT15), unphosphorylated KT15, and KT18EE (Table 2) were purchased from GLBioChem^TM^, Shanghai, China.

### NMR spectroscopy

All NMR experiments were performed on a Bruker DRX 600 MHz spectrometer equipped with an actively shielded cryoprobe. NMR data were processed using the NMRPipe and NMRDraw suite and analysed by Sparky (T.D. Goddard and D.G. Kneller, University of California, San Francisco). The chemical shifts were directly or indirectly referenced to DSS. A series of ^15^N-^1^H HSQC spectra of ^15^N-labelled Dok1 PTB domain, at 0.7 mM concentration, were acquired in the presence of different concentrations of unphosphorylated KT15, pSer745-β2, pSer756-β2 and pS756-KT15. The stock solutions of the above mentioned peptides were prepared in the same buffer (100 mM NaCl, 2 mM DTT, 50 mM sodium phosphate, pH 6.0) used for the Dok1 PTB domain NMR experiments, ^15^N-^1^H HSQC spectra were recorded at different protein:peptide molar ratios (1:0.1, 1:0.2, 1:0.3, 1:0.5, 1:1, 1:1.5, 1:2, 1:2.5 and 1:3) for all of the above mentioned peptides except for the unphosphorylated KT15. For the titration experiment using Dok1 PTB domain and the unphosphorylated KT15, the protein:peptide molar ratios used were 1:0.5, 1:1, 1:2, 1:3, 1:4, 1:5, and 1:6. Titration experiments were also performed using Dok1 PTB domain and Ser to Glu substituted KT18 peptide. ^15^N-^1^H HSQC spectra were recorded at different protein:peptide molar ratios (1:0.1, 1:0.2, 1:0.3, 1:0.5, 1:1, 1:1.5, 1:2, 1:3) for Ser to Glu substituted β2 tail peptide. ^1^H-^15^N HSQC spectra of all samples were recorded after 20 min of equilibration. Ligand induced chemical shift changes in ^15^N and HN resonances were determined using the following equation which is designated as chemical shift perturbation (CSP), Δ(H,N) = [{(Δ_H_)(W_H_)}^2^ + {(Δ_N_)(W_N_)}^2^]^1/2^ where W_H_ and W_N_ are weighting factors for ^1^H and ^15^N chemical shifts, respectively (W_H_ = 1, W_N_ = 0.154) and Δ_H_ and Δ_N_ are the chemical shift difference for HN and ^15^N, respectively^12^,^14^. The K_d_ value was calculated by using the depletion model for ligand binding which follows the equation; ∆(H,N) = ∆(H,N)_max_ [{[L] + [U] + K_d_} − [{[L] + [U] + K_d_}^2^ − 4[L][U]]^1/2^] ÷ 2[L], where ∆(H,N) and ∆(H,N)_max_ are the weighted chemical shift and weighted chemical shift at saturation, K_d_ represents the dissociation constant, and [L] and [U] are the concentrations of protein and unlabelled peptides, respectively. Well resolved ^15^N-^1^H HSQC peaks with significant chemical shift changes were used to fit the above equation using the program OriginPro 9.0 in order to determine K_d_ values. The dissociation constant is presented as K_d_ ± S.E (standard error). The error bar indicates the standard deviation for each titration point.

### trNOESY and STD NMR experiments

pS756-KT15 peptide at a concentration of 0.6 mM was titrated against different concentrations of Dok1 PTB domain. All signals of the pS756-KT15 peptide were broadened at protein:peptide molar ratio of ~1:30. Thus, 2D trNOESY experiments were performed with a mixing time of 150 ms. 2D TOCSY spectra were acquired at 50 ms mixing time. The spectral width was 12 ppm in both dimensions. Spectra were processed using the Topspin program suite followed by analysis using Sparky. For STD-NMR, 0.6 mM of pS756-KT15 or unphosphorylated KT15 peptide was dissolved in 600 μl of D_2_O (pH 6.2). The Dok1 PTB domain (20 μM) was buffer exchanged in 99.9% D_2_O. STD-NMR spectra were recorded with 512 scans and selective saturation of protein resonances at −0.7 ppm (40 ppm for reference spectra) using a series of 40 Gaussian-shaped pulses (49 ms, 1 ms delay between pulses), for a total saturation time of 2 s.

### ^31^P NMR experiments

^31^P NMR spectra of pS756-KT15 were recorded on a Bruker DRX 400 spectrometer at 298 K. Data acquisition and processing were performed with the Topspin software (BRUKER) suite. 1-D ^31^P NMR spectra of pS756-KT15 at 0.2 mM in water (pH 6.2) were recorded in the presence of different concentrations of Dok1 PTB domain. The Dok1 PTB stock solution used was prepared in unbuffered water (pH 6.2). The pH of each sample was adjusted to 6.2 after addition of protein solutions.

### Docking of Dok1 PTB domain with pS756-KT15 peptide

Based on the conformation of NAK peptide (pdb accession code, 1DDM.pdb), a structural model of pS756-KT15 peptide was built using INSIGHTII (Accelrys Inc.) software. The model structure of pS756KT15 was energy minimized by conjugate gradient energy minimization protocol. The model of pS756-KT15/Dok1 complex was derived using HADDOCK program (http://haddock.science.uu.nl/) based om chemical shift changes of the PTB domain. The model structure was validated using ADIT Validation Server from the Protein Data Bank (PDB) and coordinates of the complex are provided in supporting materials.

## Additional Information

**How to cite this article**: Gupta, S. *et al.* An Alternative Phosphorylation Switch in Integrin β2 (CD18) Tail for Dok1 Binding. *Sci. Rep.* **5**, 11630; doi: 10.1038/srep11630 (2015).

## Supplementary Material

Supplementary Information

## Figures and Tables

**Table 1:** Comparison of amino acid sequences of integrin cytoplasmic tails highlighting the NxxY/F motif (blue) and potential phosphorylation of Ser residues (red).

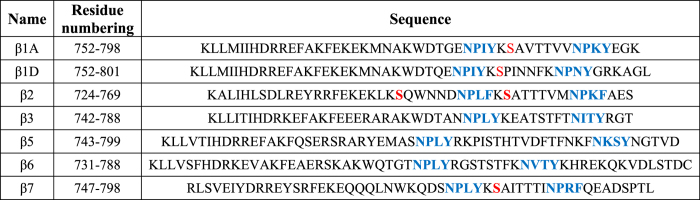

**Table 2:** Dissociation constant (K_d_) values determined for integrin β2 tail and Dok1 interactions*.

**β2 tail peptides**	**K**_**d**_ **(mM)**
pSer745-β2	0.215 ± 0.02
pSer756-β2	0.098 ± 0.02
KT15	16 ± 0.3
pSer756-KT15	0.189 ± 0.03
KT18EE	8.49 ± 0.2

^*^Full-length β2 tail phosphorylated at residue Ser745 (pSer745-β2) or Ser756 (pSer756-β2), KT15 (K^744^SQWNNDNPLFKSAT^758^), pSer756-KT15 (KSQWNNDNPLFKpSAT), KT18EE (K^742^LKEQWNNDNPLFKEATT^759^).
