# Supplementary material for: An Alternative Phosphorylation Switch in Integrin β2 (CD18) Tail for Dok1 Binding
Source: Sci Rep. 2015 Jun 25;5:11630. doi: 10.1038/srep11630 (PMC4479986; doi:10.1038/srep11630)
Supplement: Supplementary Information [file srep11630-s1.doc]

**An Alternative Phosphorylation Switch in Integrin β2 (CD18) Tail for Dok1 Binding**

Sebanti Gupta, Joel Chia-Yeong Chit, Chen Feng, Anirban Bhunia, Suet-Mien Tan*, and Surajit Bhattacharjya*

School of Biological Sciences, Nanyang Technological University, 60 Nanyang Drive, Singapore 637551.

**Figure S1**

**
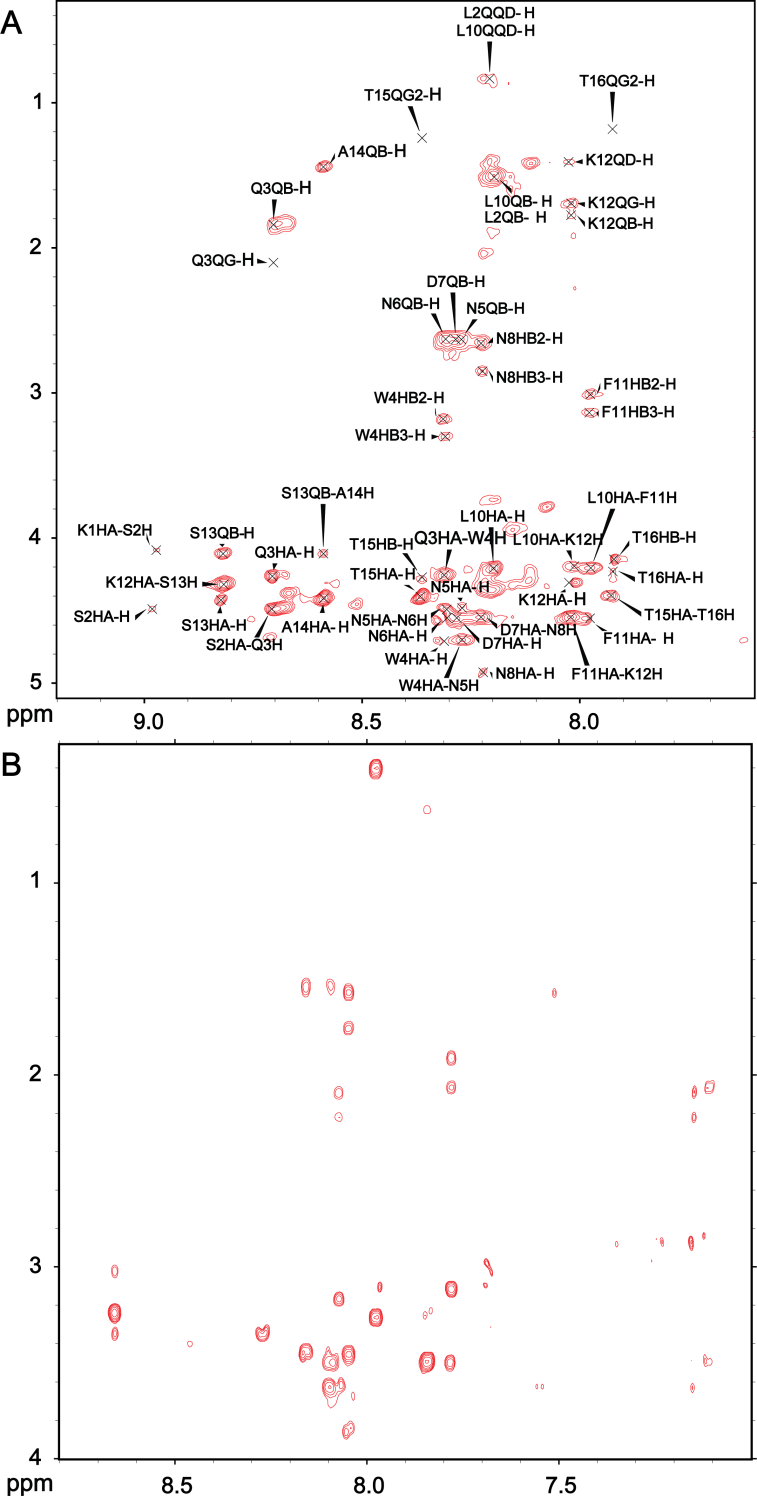
**

**Figure S1:** A section of tr-NOESY spectrum (top panel) of pSer756-KT15 peptide showing NOE correlations among downfield shifted aromatic/amide resonances (7.5-9.2 ppm) with up-field shifted aliphatic resonances (0.7-5 ppm). The presence of sequential and intra-residue NOEs indicate that the pSer756-KT15 β2 peptide predominantly adopts extended conformations in complex with the Dok1 PTB domain. (lower panel) Section of NOESY spectrum of pSer756-KT15 peptide in free solution showing lesser number NOEs, indicating random conformations.

**Figure S2**


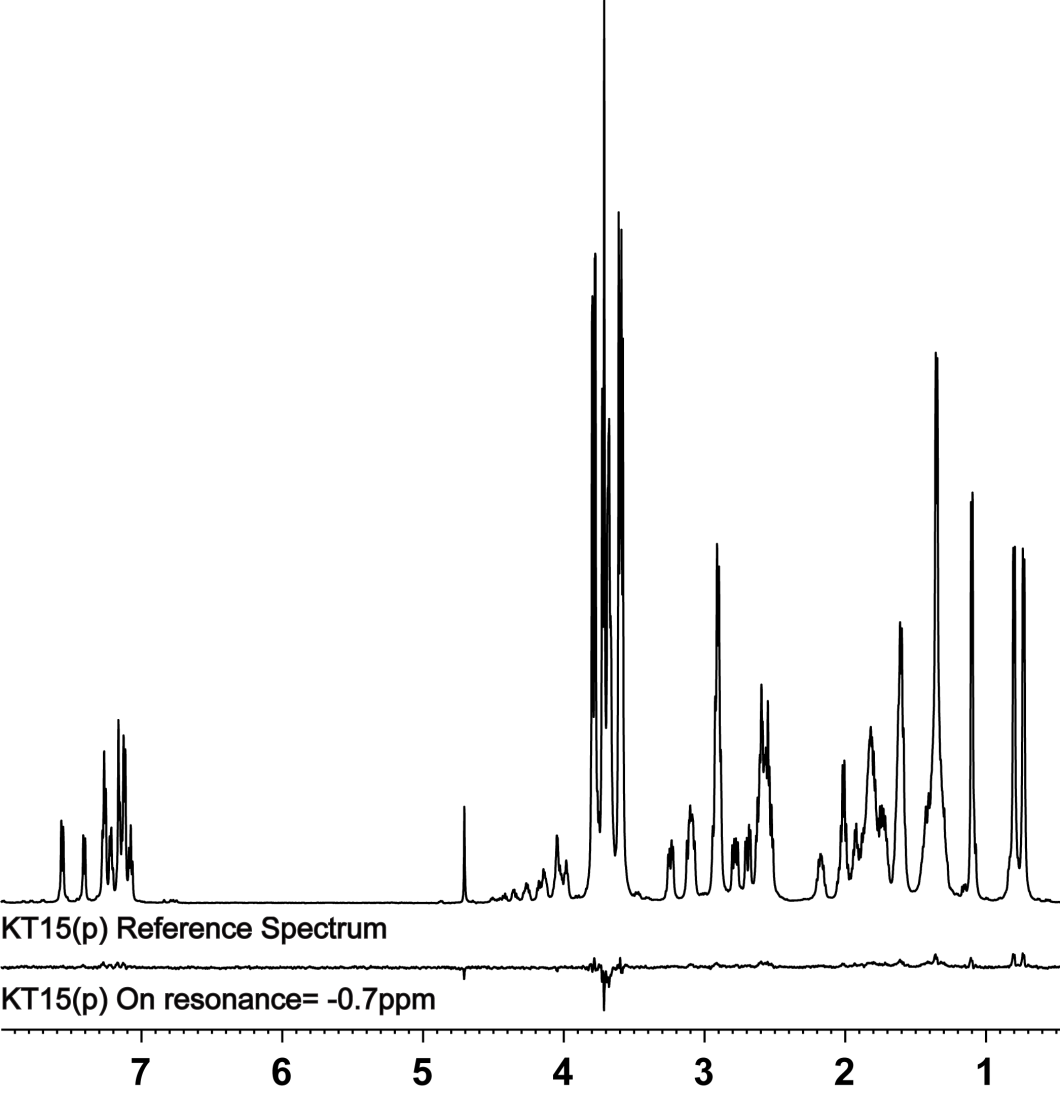


**Figure S2:** STD spectrum of pSer756-KT15 peptide in the absence of Dok1 PTB domain (at lower panel). The reference spectrum of unphosphorylated KT15 peptide has been shown at the upper panel.

**Figure S3**

**
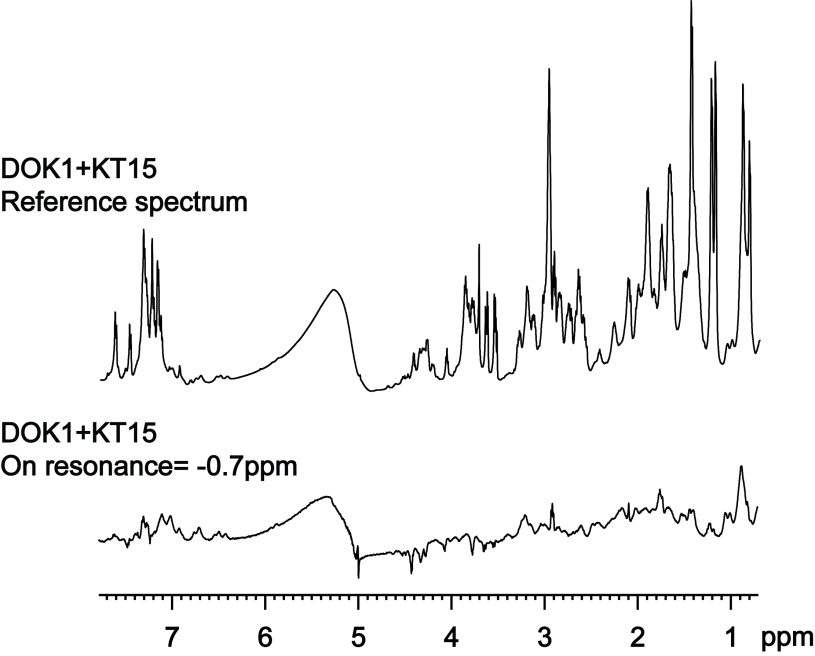
**

**Figure S3:** STD spectrum of unphosphorylated KT15 peptide in the presence of Dok1 PTB domain (at lower panel). The reference spectrum of unphosphorylated KT15 peptide has been shown at the upper panel.

**Figure S4**


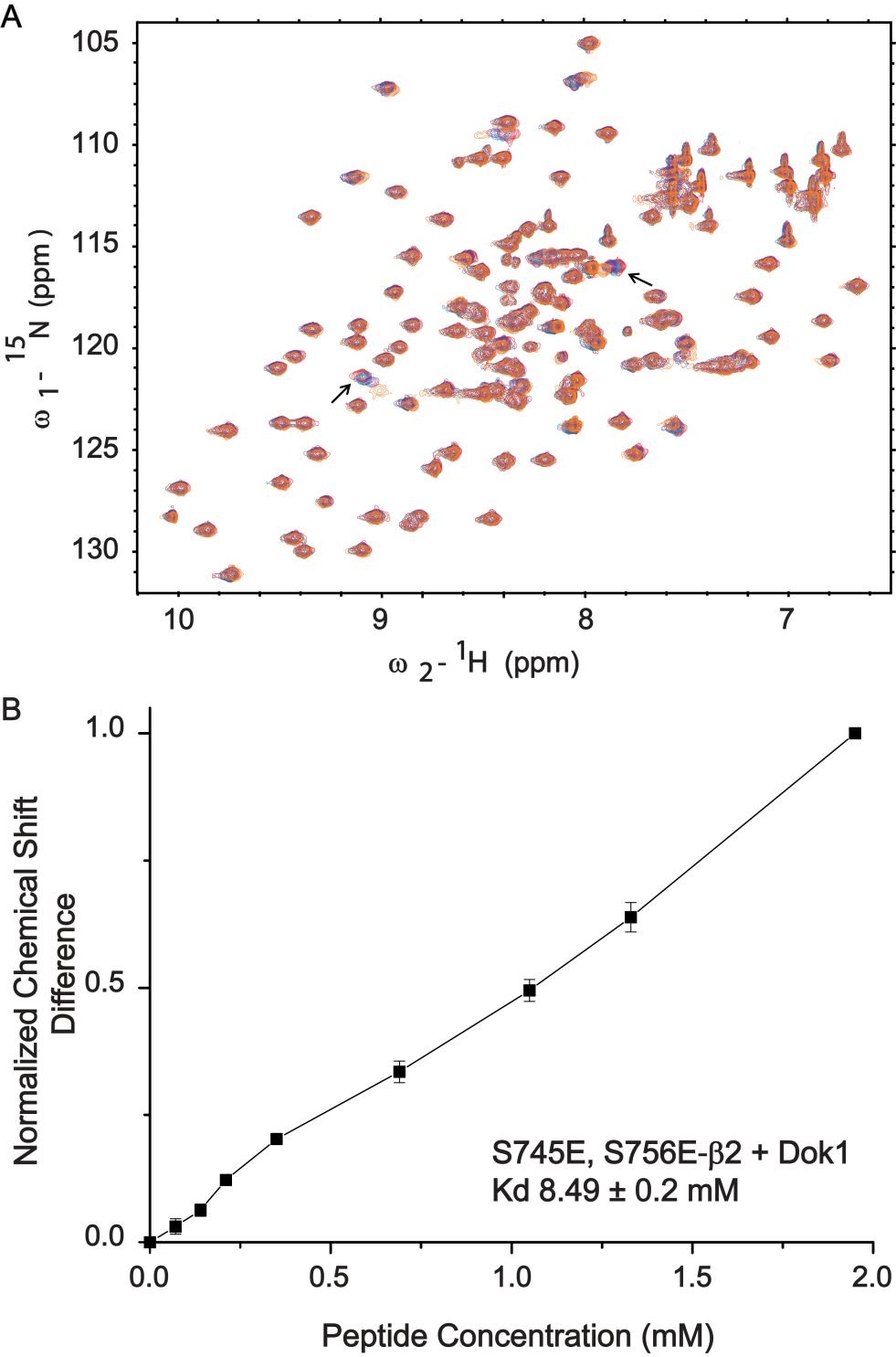


**Figure S4:** (Panel A) ^15^N-^1^H HSQC spectra of the Dok1 PTB domain at 0 mM (red contour), 0.4 mM (cyan contour), 1 mM (violet contour) and 2 mM (orange contour) concentrations of S745E, S756E substituted β2 tail. Glu substituted β2 tail binds to the PTB domain of Dok1 with low affinity (panel B).

Pdb coordinates for the complex of Dok1 pKT15 peptide

Pdb coordinates of the PTB domain of Dok1 and b2 peptide phosphorylated peptide KT15

ATOM 1 CB MET A 4 -4.554 10.838 -8.059 1.00 10.00 A

ATOM 2 CG MET A 4 -4.834 12.156 -7.356 1.00 10.00 A

ATOM 3 SD MET A 4 -6.597 12.524 -7.240 1.00 10.00 A

ATOM 4 CE MET A 4 -6.550 14.160 -6.508 1.00 10.00 A

ATOM 5 C MET A 4 -4.021 9.644 -10.196 1.00 10.00 A

ATOM 6 O MET A 4 -3.128 9.731 -11.038 1.00 10.00 A

ATOM 7 N MET A 4 -6.053 11.060 -10.033 1.00 10.00 A

ATOM 8 HT1 MET A 4 -6.618 10.233 -9.757 1.00 10.00 A

ATOM 9 HT2 MET A 4 -6.087 11.151 -11.068 1.00 10.00 A

ATOM 10 HT3 MET A 4 -6.469 11.912 -9.609 1.00 10.00 A

ATOM 11 CA MET A 4 -4.648 10.894 -9.586 1.00 10.00 A

ATOM 12 N GLY A 5 -4.486 8.479 -9.766 1.00 10.00 A

ATOM 13 HN GLY A 5 -5.199 8.461 -9.094 1.00 10.00 A

ATOM 14 CA GLY A 5 -3.956 7.234 -10.282 1.00 10.00 A

ATOM 15 C GLY A 5 -5.037 6.196 -10.503 1.00 10.00 A

ATOM 16 O GLY A 5 -6.226 6.521 -10.525 1.00 10.00 A

ATOM 17 N SER A 6 -4.628 4.947 -10.653 1.00 10.00 A

ATOM 18 HN SER A 6 -3.670 4.749 -10.595 1.00 10.00 A

ATOM 19 CA SER A 6 -5.562 3.858 -10.882 1.00 10.00 A

ATOM 20 CB SER A 6 -4.873 2.737 -11.659 1.00 10.00 A

ATOM 21 OG SER A 6 -4.205 3.252 -12.799 1.00 10.00 A

ATOM 22 HG SER A 6 -4.287 4.212 -12.809 1.00 10.00 A

ATOM 23 C SER A 6 -6.120 3.320 -9.570 1.00 10.00 A

ATOM 24 O SER A 6 -5.390 3.160 -8.591 1.00 10.00 A

ATOM 25 N GLN A 7 -7.416 3.050 -9.558 1.00 10.00 A

ATOM 26 HN GLN A 7 -7.944 3.202 -10.373 1.00 10.00 A

ATOM 27 CA GLN A 7 -8.080 2.525 -8.376 1.00 10.00 A

ATOM 28 CB GLN A 7 -9.334 3.344 -8.063 1.00 10.00 A

ATOM 29 CG GLN A 7 -9.116 4.847 -8.114 1.00 10.00 A

ATOM 30 CD GLN A 7 -10.147 5.629 -7.325 1.00 10.00 A

ATOM 31 OE1 GLN A 7 -9.838 6.677 -6.756 1.00 10.00 A

ATOM 32 NE2 GLN A 7 -11.377 5.138 -7.288 1.00 10.00 A

ATOM 33 HE21 GLN A 7 -11.562 4.299 -7.771 1.00 10.00 A

ATOM 34 HE22 GLN A 7 -12.058 5.632 -6.787 1.00 10.00 A

ATOM 35 C GLN A 7 -8.453 1.066 -8.604 1.00 10.00 A

ATOM 36 O GLN A 7 -8.975 0.714 -9.663 1.00 10.00 A

ATOM 37 N PHE A 8 -8.176 0.219 -7.625 1.00 10.00 A

ATOM 38 HN PHE A 8 -7.755 0.553 -6.800 1.00 10.00 A

ATOM 39 CA PHE A 8 -8.483 -1.198 -7.744 1.00 10.00 A

ATOM 40 CB PHE A 8 -7.196 -2.013 -7.912 1.00 10.00 A

ATOM 41 CG PHE A 8 -6.440 -1.719 -9.174 1.00 10.00 A

ATOM 42 CD1 PHE A 8 -5.250 -1.009 -9.134 1.00 10.00 A

ATOM 43 CD2 PHE A 8 -6.916 -2.153 -10.399 1.00 10.00 A

ATOM 44 CE1 PHE A 8 -4.551 -0.737 -10.294 1.00 10.00 A

ATOM 45 CE2 PHE A 8 -6.223 -1.884 -11.562 1.00 10.00 A

ATOM 46 CZ PHE A 8 -5.039 -1.176 -11.509 1.00 10.00 A

ATOM 47 C PHE A 8 -9.252 -1.706 -6.532 1.00 10.00 A

ATOM 48 O PHE A 8 -9.073 -1.211 -5.416 1.00 10.00 A

ATOM 49 N TRP A 9 -10.115 -2.686 -6.768 1.00 10.00 A

ATOM 50 HN TRP A 9 -10.226 -3.007 -7.687 1.00 10.00 A

ATOM 51 CA TRP A 9 -10.901 -3.301 -5.713 1.00 10.00 A

ATOM 52 CB TRP A 9 -12.143 -3.966 -6.319 1.00 10.00 A

ATOM 53 CG TRP A 9 -13.183 -4.370 -5.319 1.00 10.00 A

ATOM 54 CD1 TRP A 9 -13.215 -5.522 -4.585 1.00 10.00 A

ATOM 55 CD2 TRP A 9 -14.354 -3.629 -4.950 1.00 10.00 A

ATOM 56 NE1 TRP A 9 -14.331 -5.545 -3.784 1.00 10.00 A

ATOM 57 HE1 TRP A 9 -14.574 -6.271 -3.168 1.00 10.00 A

ATOM 58 CE2 TRP A 9 -15.046 -4.393 -3.988 1.00 10.00 A

ATOM 59 CE3 TRP A 9 -14.884 -2.393 -5.336 1.00 10.00 A

ATOM 60 CZ2 TRP A 9 -16.241 -3.962 -3.411 1.00 10.00 A

ATOM 61 CZ3 TRP A 9 -16.070 -1.968 -4.762 1.00 10.00 A

ATOM 62 CH2 TRP A 9 -16.735 -2.749 -3.809 1.00 10.00 A

ATOM 63 C TRP A 9 -10.037 -4.340 -5.006 1.00 10.00 A

ATOM 64 O TRP A 9 -9.759 -5.410 -5.551 1.00 10.00 A

ATOM 65 N VAL A 10 -9.609 -4.019 -3.799 1.00 10.00 A

ATOM 66 HN VAL A 10 -9.896 -3.170 -3.399 1.00 10.00 A

ATOM 67 CA VAL A 10 -8.753 -4.912 -3.037 1.00 10.00 A

ATOM 68 CB VAL A 10 -7.403 -4.252 -2.680 1.00 10.00 A

ATOM 69 CG1 VAL A 10 -6.582 -3.996 -3.935 1.00 10.00 A

ATOM 70 CG2 VAL A 10 -7.615 -2.961 -1.901 1.00 10.00 A

ATOM 71 C VAL A 10 -9.430 -5.392 -1.762 1.00 10.00 A

ATOM 72 O VAL A 10 -10.308 -4.720 -1.215 1.00 10.00 A

ATOM 73 N THR A 11 -9.022 -6.560 -1.302 1.00 10.00 A

ATOM 74 HN THR A 11 -8.341 -7.058 -1.801 1.00 10.00 A

ATOM 75 CA THR A 11 -9.568 -7.133 -0.087 1.00 10.00 A

ATOM 76 CB THR A 11 -10.119 -8.548 -0.337 1.00 10.00 A

ATOM 77 OG1 THR A 11 -10.379 -8.710 -1.741 1.00 10.00 A

ATOM 78 HG1 THR A 11 -10.063 -9.578 -2.028 1.00 10.00 A

ATOM 79 CG2 THR A 11 -11.417 -8.743 0.427 1.00 10.00 A

ATOM 80 C THR A 11 -8.503 -7.161 1.008 1.00 10.00 A

ATOM 81 O THR A 11 -7.459 -7.803 0.861 1.00 10.00 A

ATOM 82 N SER A 12 -8.768 -6.448 2.092 1.00 10.00 A

ATOM 83 HN SER A 12 -9.622 -5.965 2.146 1.00 10.00 A

ATOM 84 CA SER A 12 -7.837 -6.357 3.206 1.00 10.00 A

ATOM 85 CB SER A 12 -8.243 -5.197 4.114 1.00 10.00 A

ATOM 86 OG SER A 12 -8.676 -4.086 3.347 1.00 10.00 A

ATOM 87 HG SER A 12 -9.551 -4.266 2.986 1.00 10.00 A

ATOM 88 C SER A 12 -7.785 -7.650 4.014 1.00 10.00 A

ATOM 89 O SER A 12 -8.820 -8.173 4.436 1.00 10.00 A

ATOM 90 N GLN A 13 -6.577 -8.160 4.225 1.00 10.00 A

ATOM 91 HN GLN A 13 -5.790 -7.705 3.850 1.00 10.00 A

ATOM 92 CA GLN A 13 -6.387 -9.375 5.001 1.00 10.00 A

ATOM 93 CB GLN A 13 -5.130 -10.121 4.535 1.00 10.00 A

ATOM 94 CG GLN A 13 -4.956 -11.499 5.158 1.00 10.00 A

ATOM 95 CD GLN A 13 -3.601 -12.109 4.870 1.00 10.00 A

ATOM 96 OE1 GLN A 13 -2.653 -11.930 5.634 1.00 10.00 A

ATOM 97 NE2 GLN A 13 -3.505 -12.844 3.770 1.00 10.00 A

ATOM 98 HE21 GLN A 13 -4.306 -12.948 3.212 1.00 10.00 A

ATOM 99 HE22 GLN A 13 -2.637 -13.253 3.558 1.00 10.00 A

ATOM 100 C GLN A 13 -6.268 -9.020 6.478 1.00 10.00 A

ATOM 101 O GLN A 13 -5.828 -7.921 6.827 1.00 10.00 A

ATOM 102 N LYS A 14 -6.676 -9.937 7.340 1.00 10.00 A

ATOM 103 HN LYS A 14 -7.039 -10.785 7.002 1.00 10.00 A

ATOM 104 CA LYS A 14 -6.607 -9.716 8.773 1.00 10.00 A

ATOM 105 CB LYS A 14 -7.315 -10.841 9.532 1.00 10.00 A

ATOM 106 CG LYS A 14 -7.320 -10.661 11.043 1.00 10.00 A

ATOM 107 CD LYS A 14 -7.836 -11.904 11.751 1.00 10.00 A

ATOM 108 CE LYS A 14 -7.776 -11.753 13.263 1.00 10.00 A

ATOM 109 NZ LYS A 14 -8.858 -10.872 13.778 1.00 10.00 A

ATOM 110 HZ1 LYS A 14 -9.350 -11.335 14.568 1.00 10.00 A

ATOM 111 HZ2 LYS A 14 -9.547 -10.673 13.029 1.00 10.00 A

ATOM 112 HZ3 LYS A 14 -8.459 -9.966 14.118 1.00 10.00 A

ATOM 113 C LYS A 14 -5.157 -9.598 9.234 1.00 10.00 A

ATOM 114 O LYS A 14 -4.409 -10.574 9.225 1.00 10.00 A

ATOM 115 N THR A 15 -4.759 -8.389 9.591 1.00 10.00 A

ATOM 116 HN THR A 15 -5.390 -7.642 9.525 1.00 10.00 A

ATOM 117 CA THR A 15 -3.414 -8.125 10.073 1.00 10.00 A

ATOM 118 CB THR A 15 -2.519 -7.561 8.949 1.00 10.00 A

ATOM 119 OG1 THR A 15 -2.959 -8.076 7.685 1.00 10.00 A

ATOM 120 HG1 THR A 15 -3.416 -8.911 7.823 1.00 10.00 A

ATOM 121 CG2 THR A 15 -1.066 -7.956 9.167 1.00 10.00 A

ATOM 122 C THR A 15 -3.495 -7.114 11.214 1.00 10.00 A

ATOM 123 O THR A 15 -4.476 -6.375 11.313 1.00 10.00 A

ATOM 124 N GLU A 16 -2.481 -7.093 12.073 1.00 10.00 A

ATOM 125 HN GLU A 16 -1.740 -7.721 11.946 1.00 10.00 A

ATOM 126 CA GLU A 16 -2.441 -6.178 13.210 1.00 10.00 A

ATOM 127 CB GLU A 16 -1.084 -6.263 13.905 1.00 10.00 A

ATOM 128 CG GLU A 16 -0.982 -5.408 15.156 1.00 10.00 A

ATOM 129 CD GLU A 16 0.437 -4.975 15.445 1.00 10.00 A

ATOM 130 OE1 GLU A 16 1.347 -5.826 15.375 1.00 10.00 A

ATOM 131 OE2 GLU A 16 0.648 -3.782 15.748 1.00 10.00 A

ATOM 132 C GLU A 16 -2.714 -4.736 12.780 1.00 10.00 A

ATOM 133 O GLU A 16 -3.504 -4.026 13.408 1.00 10.00 A

ATOM 134 N ALA A 17 -2.064 -4.315 11.705 1.00 10.00 A

ATOM 135 HN ALA A 17 -1.458 -4.930 11.243 1.00 10.00 A

ATOM 136 CA ALA A 17 -2.231 -2.963 11.191 1.00 10.00 A

ATOM 137 CB ALA A 17 -1.241 -2.696 10.070 1.00 10.00 A

ATOM 138 C ALA A 17 -3.664 -2.728 10.717 1.00 10.00 A

ATOM 139 O ALA A 17 -4.273 -1.709 11.042 1.00 10.00 A

ATOM 140 N SER A 18 -4.202 -3.684 9.967 1.00 10.00 A

ATOM 141 HN SER A 18 -3.673 -4.481 9.751 1.00 10.00 A

ATOM 142 CA SER A 18 -5.559 -3.587 9.444 1.00 10.00 A

ATOM 143 CB SER A 18 -5.842 -4.786 8.539 1.00 10.00 A

ATOM 144 OG SER A 18 -4.629 -5.414 8.157 1.00 10.00 A

ATOM 145 HG SER A 18 -4.820 -6.146 7.554 1.00 10.00 A

ATOM 146 C SER A 18 -6.583 -3.531 10.575 1.00 10.00 A

ATOM 147 O SER A 18 -7.533 -2.746 10.531 1.00 10.00 A

ATOM 148 N GLU A 19 -6.375 -4.356 11.593 1.00 10.00 A

ATOM 149 HN GLU A 19 -5.593 -4.955 11.572 1.00 10.00 A

ATOM 150 CA GLU A 19 -7.277 -4.408 12.735 1.00 10.00 A

ATOM 151 CB GLU A 19 -6.958 -5.613 13.614 1.00 10.00 A

ATOM 152 CG GLU A 19 -7.210 -6.952 12.943 1.00 10.00 A

ATOM 153 CD GLU A 19 -7.012 -8.114 13.891 1.00 10.00 A

ATOM 154 OE1 GLU A 19 -5.894 -8.656 13.951 1.00 10.00 A

ATOM 155 OE2 GLU A 19 -7.975 -8.484 14.594 1.00 10.00 A

ATOM 156 C GLU A 19 -7.218 -3.121 13.553 1.00 10.00 A

ATOM 157 O GLU A 19 -8.229 -2.665 14.080 1.00 10.00 A

ATOM 158 N ARG A 20 -6.031 -2.537 13.646 1.00 10.00 A

ATOM 159 HN ARG A 20 -5.258 -2.955 13.211 1.00 10.00 A

ATOM 160 CA ARG A 20 -5.846 -1.298 14.395 1.00 10.00 A

ATOM 161 CB ARG A 20 -4.358 -0.969 14.529 1.00 10.00 A

ATOM 162 CG ARG A 20 -4.063 0.289 15.331 1.00 10.00 A

ATOM 163 CD ARG A 20 -2.571 0.455 15.576 1.00 10.00 A

ATOM 164 NE ARG A 20 -1.814 0.610 14.334 1.00 10.00 A

ATOM 165 HE ARG A 20 -2.135 1.283 13.692 1.00 10.00 A

ATOM 166 CZ ARG A 20 -0.721 -0.091 14.035 1.00 10.00 A

ATOM 167 NH1 ARG A 20 -0.258 -1.002 14.884 1.00 10.00 A

ATOM 168 HH11 ARG A 20 -0.751 -1.172 15.790 1.00 10.00 A

ATOM 169 HH12 ARG A 20 0.598 -1.553 14.655 1.00 10.00 A

ATOM 170 NH2 ARG A 20 -0.094 0.122 12.887 1.00 10.00 A

ATOM 171 HH21 ARG A 20 -0.458 0.839 12.215 1.00 10.00 A

ATOM 172 HH22 ARG A 20 0.777 -0.411 12.654 1.00 10.00 A

ATOM 173 C ARG A 20 -6.587 -0.144 13.725 1.00 10.00 A

ATOM 174 O ARG A 20 -7.148 0.723 14.395 1.00 10.00 A

ATOM 175 N CYS A 21 -6.596 -0.145 12.399 1.00 10.00 A

ATOM 176 HN CYS A 21 -6.137 -0.865 11.916 1.00 10.00 A

ATOM 177 CA CYS A 21 -7.272 0.901 11.641 1.00 10.00 A

ATOM 178 CB CYS A 21 -6.677 1.006 10.236 1.00 10.00 A

ATOM 179 SG CYS A 21 -4.871 1.091 10.206 1.00 10.00 A

ATOM 180 HG CYS A 21 -4.405 -0.109 10.529 1.00 10.00 A

ATOM 181 C CYS A 21 -8.776 0.642 11.567 1.00 10.00 A

ATOM 182 O CYS A 21 -9.558 1.541 11.267 1.00 10.00 A

ATOM 183 N GLY A 22 -9.170 -0.593 11.848 1.00 10.00 A

ATOM 184 HN GLY A 22 -8.502 -1.269 12.084 1.00 10.00 A

ATOM 185 CA GLY A 22 -10.575 -0.948 11.813 1.00 10.00 A

ATOM 186 C GLY A 22 -11.058 -1.231 10.405 1.00 10.00 A

ATOM 187 O GLY A 22 -12.150 -0.811 10.017 1.00 10.00 A

ATOM 188 N LEU A 23 -10.241 -1.939 9.637 1.00 10.00 A

ATOM 189 HN LEU A 23 -9.383 -2.242 10.004 1.00 10.00 A

ATOM 190 CA LEU A 23 -10.586 -2.280 8.266 1.00 10.00 A

ATOM 191 CB LEU A 23 -9.323 -2.538 7.437 1.00 10.00 A

ATOM 192 CG LEU A 23 -8.289 -1.409 7.399 1.00 10.00 A

ATOM 193 CD1 LEU A 23 -7.111 -1.789 6.515 1.00 10.00 A

ATOM 194 CD2 LEU A 23 -8.923 -0.112 6.920 1.00 10.00 A

ATOM 195 C LEU A 23 -11.493 -3.504 8.227 1.00 10.00 A

ATOM 196 O LEU A 23 -11.237 -4.502 8.906 1.00 10.00 A

ATOM 197 N GLN A 24 -12.555 -3.421 7.440 1.00 10.00 A

ATOM 198 HN GLN A 24 -12.716 -2.586 6.938 1.00 10.00 A

ATOM 199 CA GLN A 24 -13.494 -4.528 7.309 1.00 10.00 A

ATOM 200 CB GLN A 24 -14.888 -4.040 6.909 1.00 10.00 A

ATOM 201 CG GLN A 24 -15.610 -3.257 7.991 1.00 10.00 A

ATOM 202 CD GLN A 24 -15.443 -1.764 7.833 1.00 10.00 A

ATOM 203 OE1 GLN A 24 -15.391 -1.247 6.717 1.00 10.00 A

ATOM 204 NE2 GLN A 24 -15.340 -1.060 8.945 1.00 10.00 A

ATOM 205 HE21 GLN A 24 -15.373 -1.537 9.802 1.00 10.00 A

ATOM 206 HE22 GLN A 24 -15.230 -0.090 8.867 1.00 10.00 A

ATOM 207 C GLN A 24 -12.985 -5.573 6.319 1.00 10.00 A

ATOM 208 O GLN A 24 -12.594 -6.670 6.712 1.00 10.00 A

ATOM 209 N GLY A 25 -12.983 -5.231 5.036 1.00 10.00 A

ATOM 210 HN GLY A 25 -13.299 -4.343 4.770 1.00 10.00 A

ATOM 211 CA GLY A 25 -12.518 -6.170 4.030 1.00 10.00 A

ATOM 212 C GLY A 25 -12.359 -5.550 2.656 1.00 10.00 A

ATOM 213 O GLY A 25 -11.255 -5.158 2.271 1.00 10.00 A

ATOM 214 N SER A 26 -13.456 -5.464 1.919 1.00 10.00 A

ATOM 215 HN SER A 26 -14.305 -5.791 2.285 1.00 10.00 A

ATOM 216 CA SER A 26 -13.444 -4.902 0.572 1.00 10.00 A

ATOM 217 CB SER A 26 -14.754 -5.249 -0.136 1.00 10.00 A

ATOM 218 OG SER A 26 -15.239 -6.516 0.291 1.00 10.00 A

ATOM 219 HG SER A 26 -16.198 -6.541 0.190 1.00 10.00 A

ATOM 220 C SER A 26 -13.239 -3.386 0.587 1.00 10.00 A

ATOM 221 O SER A 26 -13.983 -2.657 1.244 1.00 10.00 A

ATOM 222 N TYR A 27 -12.225 -2.920 -0.138 1.00 10.00 A

ATOM 223 HN TYR A 27 -11.660 -3.555 -0.641 1.00 10.00 A

ATOM 224 CA TYR A 27 -11.919 -1.495 -0.217 1.00 10.00 A

ATOM 225 CB TYR A 27 -10.873 -1.103 0.832 1.00 10.00 A

ATOM 226 CG TYR A 27 -11.391 -1.051 2.249 1.00 10.00 A

ATOM 227 CD1 TYR A 27 -11.063 -2.043 3.158 1.00 10.00 A

ATOM 228 CD2 TYR A 27 -12.207 -0.011 2.677 1.00 10.00 A

ATOM 229 CE1 TYR A 27 -11.529 -2.006 4.454 1.00 10.00 A

ATOM 230 CE2 TYR A 27 -12.680 0.034 3.976 1.00 10.00 A

ATOM 231 CZ TYR A 27 -12.336 -0.968 4.858 1.00 10.00 A

ATOM 232 OH TYR A 27 -12.797 -0.933 6.150 1.00 10.00 A

ATOM 233 HH TYR A 27 -13.740 -0.719 6.152 1.00 10.00 A

ATOM 234 C TYR A 27 -11.392 -1.141 -1.600 1.00 10.00 A

ATOM 235 O TYR A 27 -11.087 -2.024 -2.401 1.00 10.00 A

ATOM 236 N ILE A 28 -11.288 0.151 -1.877 1.00 10.00 A

ATOM 237 HN ILE A 28 -11.555 0.812 -1.200 1.00 10.00 A

ATOM 238 CA ILE A 28 -10.781 0.618 -3.157 1.00 10.00 A

ATOM 239 CB ILE A 28 -11.794 1.532 -3.885 1.00 10.00 A

ATOM 240 CG1 ILE A 28 -13.094 0.765 -4.158 1.00 10.00 A

ATOM 241 CG2 ILE A 28 -11.202 2.059 -5.186 1.00 10.00 A

ATOM 242 CD1 ILE A 28 -14.154 1.574 -4.877 1.00 10.00 A

ATOM 243 C ILE A 28 -9.461 1.355 -2.951 1.00 10.00 A

ATOM 244 O ILE A 28 -9.426 2.451 -2.389 1.00 10.00 A

ATOM 245 N LEU A 29 -8.378 0.739 -3.389 1.00 10.00 A

ATOM 246 HN LEU A 29 -8.471 -0.131 -3.838 1.00 10.00 A

ATOM 247 CA LEU A 29 -7.056 1.330 -3.245 1.00 10.00 A

ATOM 248 CB LEU A 29 -6.032 0.256 -2.863 1.00 10.00 A

ATOM 249 CG LEU A 29 -4.588 0.729 -2.665 1.00 10.00 A

ATOM 250 CD1 LEU A 29 -4.489 1.693 -1.492 1.00 10.00 A

ATOM 251 CD2 LEU A 29 -3.657 -0.459 -2.466 1.00 10.00 A

ATOM 252 C LEU A 29 -6.640 2.021 -4.531 1.00 10.00 A

ATOM 253 O LEU A 29 -6.711 1.435 -5.610 1.00 10.00 A

ATOM 254 N ARG A 30 -6.215 3.268 -4.420 1.00 10.00 A

ATOM 255 HN ARG A 30 -6.179 3.690 -3.531 1.00 10.00 A

ATOM 256 CA ARG A 30 -5.790 4.024 -5.587 1.00 10.00 A

ATOM 257 CB ARG A 30 -6.612 5.306 -5.754 1.00 10.00 A

ATOM 258 CG ARG A 30 -6.959 6.006 -4.452 1.00 10.00 A

ATOM 259 CD ARG A 30 -6.699 7.501 -4.528 1.00 10.00 A

ATOM 260 NE ARG A 30 -7.717 8.210 -5.302 1.00 10.00 A

ATOM 261 HE ARG A 30 -8.419 7.666 -5.734 1.00 10.00 A

ATOM 262 CZ ARG A 30 -7.741 9.537 -5.449 1.00 10.00 A

ATOM 263 NH1 ARG A 30 -6.793 10.291 -4.894 1.00 10.00 A

ATOM 264 HH11 ARG A 30 -6.013 9.848 -4.337 1.00 10.00 A

ATOM 265 HH12 ARG A 30 -6.813 11.324 -5.008 1.00 10.00 A

ATOM 266 NH2 ARG A 30 -8.700 10.111 -6.166 1.00 10.00 A

ATOM 267 HH21 ARG A 30 -9.435 9.528 -6.622 1.00 10.00 A

ATOM 268 HH22 ARG A 30 -8.720 11.146 -6.282 1.00 10.00 A

ATOM 269 C ARG A 30 -4.307 4.349 -5.523 1.00 10.00 A

ATOM 270 O ARG A 30 -3.844 4.985 -4.576 1.00 10.00 A

ATOM 271 N VAL A 31 -3.571 3.899 -6.526 1.00 10.00 A

ATOM 272 HN VAL A 31 -4.007 3.396 -7.249 1.00 10.00 A

ATOM 273 CA VAL A 31 -2.138 4.148 -6.598 1.00 10.00 A

ATOM 274 CB VAL A 31 -1.395 3.032 -7.385 1.00 10.00 A

ATOM 275 CG1 VAL A 31 -2.102 2.698 -8.691 1.00 10.00 A

ATOM 276 CG2 VAL A 31 0.060 3.398 -7.636 1.00 10.00 A

ATOM 277 C VAL A 31 -1.885 5.516 -7.224 1.00 10.00 A

ATOM 278 O VAL A 31 -2.126 5.722 -8.417 1.00 10.00 A

ATOM 279 N GLU A 32 -1.436 6.458 -6.407 1.00 10.00 A

ATOM 280 HN GLU A 32 -1.270 6.238 -5.463 1.00 10.00 A

ATOM 281 CA GLU A 32 -1.172 7.807 -6.875 1.00 10.00 A

ATOM 282 CB GLU A 32 -1.633 8.841 -5.844 1.00 10.00 A

ATOM 283 CG GLU A 32 -3.096 8.693 -5.453 1.00 10.00 A

ATOM 284 CD GLU A 32 -3.592 9.818 -4.568 1.00 10.00 A

ATOM 285 OE1 GLU A 32 -4.490 9.573 -3.735 1.00 10.00 A

ATOM 286 OE2 GLU A 32 -3.103 10.956 -4.715 1.00 10.00 A

ATOM 287 C GLU A 32 0.297 7.990 -7.241 1.00 10.00 A

ATOM 288 O GLU A 32 1.045 7.017 -7.345 1.00 10.00 A

ATOM 289 N ALA A 33 0.710 9.237 -7.415 1.00 10.00 A

ATOM 290 HN ALA A 33 0.080 9.974 -7.275 1.00 10.00 A

ATOM 291 CA ALA A 33 2.080 9.536 -7.800 1.00 10.00 A

ATOM 292 CB ALA A 33 2.137 10.857 -8.556 1.00 10.00 A

ATOM 293 C ALA A 33 3.035 9.565 -6.610 1.00 10.00 A

ATOM 294 O ALA A 33 4.214 9.254 -6.755 1.00 10.00 A

ATOM 295 N GLU A 34 2.533 9.936 -5.439 1.00 10.00 A

ATOM 296 HN GLU A 34 1.582 10.175 -5.369 1.00 10.00 A

ATOM 297 CA GLU A 34 3.380 10.021 -4.258 1.00 10.00 A

ATOM 298 CB GLU A 34 3.758 11.479 -3.962 1.00 10.00 A

ATOM 299 CG GLU A 34 2.747 12.516 -4.439 1.00 10.00 A

ATOM 300 CD GLU A 34 1.377 12.347 -3.813 1.00 10.00 A

ATOM 301 OE1 GLU A 34 1.097 13.015 -2.797 1.00 10.00 A

ATOM 302 OE2 GLU A 34 0.574 11.547 -4.332 1.00 10.00 A

ATOM 303 C GLU A 34 2.767 9.364 -3.025 1.00 10.00 A

ATOM 304 O GLU A 34 3.326 9.460 -1.929 1.00 10.00 A

ATOM 305 N LYS A 35 1.637 8.686 -3.185 1.00 10.00 A

ATOM 306 HN LYS A 35 1.231 8.616 -4.076 1.00 10.00 A

ATOM 307 CA LYS A 35 0.994 8.038 -2.049 1.00 10.00 A

ATOM 308 CB LYS A 35 0.365 9.088 -1.123 1.00 10.00 A

ATOM 309 CG LYS A 35 -0.775 9.878 -1.747 1.00 10.00 A

ATOM 310 CD LYS A 35 -1.110 11.109 -0.922 1.00 10.00 A

ATOM 311 CE LYS A 35 -2.141 11.984 -1.618 1.00 10.00 A

ATOM 312 NZ LYS A 35 -1.718 12.376 -2.991 1.00 10.00 A

ATOM 313 HZ1 LYS A 35 -1.984 13.362 -3.180 1.00 10.00 A

ATOM 314 HZ2 LYS A 35 -2.186 11.764 -3.699 1.00 10.00 A

ATOM 315 HZ3 LYS A 35 -0.682 12.280 -3.097 1.00 10.00 A

ATOM 316 C LYS A 35 -0.070 7.031 -2.471 1.00 10.00 A

ATOM 317 O LYS A 35 -0.592 7.081 -3.583 1.00 10.00 A

ATOM 318 N LEU A 36 -0.361 6.110 -1.568 1.00 10.00 A

ATOM 319 HN LEU A 36 0.138 6.107 -0.719 1.00 10.00 A

ATOM 320 CA LEU A 36 -1.381 5.100 -1.779 1.00 10.00 A

ATOM 321 CB LEU A 36 -0.841 3.710 -1.421 1.00 10.00 A

ATOM 322 CG LEU A 36 0.186 3.111 -2.386 1.00 10.00 A

ATOM 323 CD1 LEU A 36 0.841 1.880 -1.779 1.00 10.00 A

ATOM 324 CD2 LEU A 36 -0.473 2.766 -3.714 1.00 10.00 A

ATOM 325 C LEU A 36 -2.560 5.437 -0.884 1.00 10.00 A

ATOM 326 O LEU A 36 -2.489 5.278 0.334 1.00 10.00 A

ATOM 327 N THR A 37 -3.624 5.941 -1.476 1.00 10.00 A

ATOM 328 HN THR A 37 -3.624 6.063 -2.450 1.00 10.00 A

ATOM 329 CA THR A 37 -4.794 6.317 -0.709 1.00 10.00 A

ATOM 330 CB THR A 37 -5.404 7.633 -1.230 1.00 10.00 A

ATOM 331 OG1 THR A 37 -4.389 8.645 -1.285 1.00 10.00 A

ATOM 332 HG1 THR A 37 -4.356 9.022 -2.182 1.00 10.00 A

ATOM 333 CG2 THR A 37 -6.532 8.102 -0.323 1.00 10.00 A

ATOM 334 C THR A 37 -5.851 5.218 -0.714 1.00 10.00 A

ATOM 335 O THR A 37 -6.225 4.704 -1.769 1.00 10.00 A

ATOM 336 N LEU A 38 -6.307 4.850 0.471 1.00 10.00 A

ATOM 337 HN LEU A 38 -5.943 5.282 1.278 1.00 10.00 A

ATOM 338 CA LEU A 38 -7.336 3.836 0.611 1.00 10.00 A

ATOM 339 CB LEU A 38 -7.096 2.990 1.865 1.00 10.00 A

ATOM 340 CG LEU A 38 -7.964 1.737 2.009 1.00 10.00 A

ATOM 341 CD1 LEU A 38 -7.656 0.739 0.902 1.00 10.00 A

ATOM 342 CD2 LEU A 38 -7.774 1.102 3.379 1.00 10.00 A

ATOM 343 C LEU A 38 -8.689 4.525 0.693 1.00 10.00 A

ATOM 344 O LEU A 38 -8.923 5.332 1.596 1.00 10.00 A

ATOM 345 N LEU A 39 -9.561 4.231 -0.255 1.00 10.00 A

ATOM 346 HN LEU A 39 -9.319 3.576 -0.950 1.00 10.00 A

ATOM 347 CA LEU A 39 -10.879 4.841 -0.288 1.00 10.00 A

ATOM 348 CB LEU A 39 -11.180 5.395 -1.685 1.00 10.00 A

ATOM 349 CG LEU A 39 -10.260 6.509 -2.194 1.00 10.00 A

ATOM 350 CD1 LEU A 39 -10.670 6.938 -3.594 1.00 10.00 A

ATOM 351 CD2 LEU A 39 -10.276 7.698 -1.246 1.00 10.00 A

ATOM 352 C LEU A 39 -11.957 3.849 0.120 1.00 10.00 A

ATOM 353 O LEU A 39 -11.906 2.671 -0.242 1.00 10.00 A

ATOM 354 N THR A 40 -12.918 4.331 0.888 1.00 10.00 A

ATOM 355 HN THR A 40 -12.889 5.277 1.158 1.00 10.00 A

ATOM 356 CA THR A 40 -14.023 3.509 1.333 1.00 10.00 A

ATOM 357 CB THR A 40 -14.178 3.549 2.874 1.00 10.00 A

ATOM 358 OG1 THR A 40 -15.200 2.636 3.294 1.00 10.00 A

ATOM 359 HG1 THR A 40 -15.754 3.057 3.959 1.00 10.00 A

ATOM 360 CG2 THR A 40 -14.515 4.952 3.359 1.00 10.00 A

ATOM 361 C THR A 40 -15.302 3.992 0.658 1.00 10.00 A

ATOM 362 O THR A 40 -15.364 5.123 0.172 1.00 10.00 A

ATOM 363 N LEU A 41 -16.311 3.139 0.614 1.00 10.00 A

ATOM 364 HN LEU A 41 -16.213 2.259 1.029 1.00 10.00 A

ATOM 365 CA LEU A 41 -17.569 3.496 -0.014 1.00 10.00 A

ATOM 366 CB LEU A 41 -18.191 2.297 -0.736 1.00 10.00 A

ATOM 367 CG LEU A 41 -17.469 1.826 -2.003 1.00 10.00 A

ATOM 368 CD1 LEU A 41 -16.506 0.689 -1.693 1.00 10.00 A

ATOM 369 CD2 LEU A 41 -18.468 1.412 -3.070 1.00 10.00 A

ATOM 370 C LEU A 41 -18.540 4.075 1.004 1.00 10.00 A

ATOM 371 O LEU A 41 -18.516 3.711 2.184 1.00 10.00 A

ATOM 372 N GLY A 42 -19.373 4.992 0.548 1.00 10.00 A

ATOM 373 HN GLY A 42 -19.316 5.262 -0.394 1.00 10.00 A

ATOM 374 CA GLY A 42 -20.345 5.612 1.417 1.00 10.00 A

ATOM 375 C GLY A 42 -21.763 5.365 0.952 1.00 10.00 A

ATOM 376 O GLY A 42 -22.121 5.723 -0.175 1.00 10.00 A

ATOM 377 N ALA A 43 -22.557 4.732 1.814 1.00 10.00 A

ATOM 378 HN ALA A 43 -22.185 4.456 2.677 1.00 10.00 A

ATOM 379 CA ALA A 43 -23.960 4.429 1.527 1.00 10.00 A

ATOM 380 CB ALA A 43 -24.785 5.709 1.509 1.00 10.00 A

ATOM 381 C ALA A 43 -24.129 3.638 0.225 1.00 10.00 A

ATOM 382 O ALA A 43 -23.217 2.935 -0.214 1.00 10.00 A

ATOM 383 N GLN A 44 -25.302 3.750 -0.385 1.00 10.00 A

ATOM 384 HN GLN A 44 -25.997 4.318 0.014 1.00 10.00 A

ATOM 385 CA GLN A 44 -25.585 3.055 -1.630 1.00 10.00 A

ATOM 386 CB GLN A 44 -27.003 2.478 -1.622 1.00 10.00 A

ATOM 387 CG GLN A 44 -27.056 0.959 -1.597 1.00 10.00 A

ATOM 388 CD GLN A 44 -26.487 0.370 -0.320 1.00 10.00 A

ATOM 389 OE1 GLN A 44 -27.210 0.147 0.652 1.00 10.00 A

ATOM 390 NE2 GLN A 44 -25.187 0.111 -0.310 1.00 10.00 A

ATOM 391 HE21 GLN A 44 -24.668 0.315 -1.115 1.00 10.00 A

ATOM 392 HE22 GLN A 44 -24.798 -0.283 0.500 1.00 10.00 A

ATOM 393 C GLN A 44 -25.391 3.987 -2.819 1.00 10.00 A

ATOM 394 O GLN A 44 -25.627 3.610 -3.965 1.00 10.00 A

ATOM 395 N SER A 45 -24.939 5.202 -2.531 1.00 10.00 A

ATOM 396 HN SER A 45 -24.762 5.438 -1.595 1.00 10.00 A

ATOM 397 CA SER A 45 -24.701 6.205 -3.556 1.00 10.00 A

ATOM 398 CB SER A 45 -24.613 7.581 -2.892 1.00 10.00 A

ATOM 399 OG SER A 45 -24.563 7.446 -1.477 1.00 10.00 A

ATOM 400 HG SER A 45 -24.417 8.312 -1.074 1.00 10.00 A

ATOM 401 C SER A 45 -23.414 5.897 -4.324 1.00 10.00 A

ATOM 402 O SER A 45 -23.145 6.484 -5.375 1.00 10.00 A

ATOM 403 N GLN A 46 -22.625 4.967 -3.779 1.00 10.00 A

ATOM 404 HN GLN A 46 -22.907 4.549 -2.935 1.00 10.00 A

ATOM 405 CA GLN A 46 -21.361 4.542 -4.384 1.00 10.00 A

ATOM 406 CB GLN A 46 -21.560 3.989 -5.801 1.00 10.00 A

ATOM 407 CG GLN A 46 -22.294 2.660 -5.865 1.00 10.00 A

ATOM 408 CD GLN A 46 -22.216 2.024 -7.238 1.00 10.00 A

ATOM 409 OE1 GLN A 46 -21.321 1.228 -7.523 1.00 10.00 A

ATOM 410 NE2 GLN A 46 -23.148 2.377 -8.108 1.00 10.00 A

ATOM 411 HE21 GLN A 46 -23.832 3.020 -7.823 1.00 10.00 A

ATOM 412 HE22 GLN A 46 -23.115 1.985 -9.005 1.00 10.00 A

ATOM 413 C GLN A 46 -20.315 5.654 -4.387 1.00 10.00 A

ATOM 414 O GLN A 46 -19.497 5.746 -5.303 1.00 10.00 A

ATOM 415 N ILE A 47 -20.341 6.497 -3.366 1.00 10.00 A

ATOM 416 HN ILE A 47 -21.019 6.387 -2.668 1.00 10.00 A

ATOM 417 CA ILE A 47 -19.374 7.581 -3.260 1.00 10.00 A

ATOM 418 CB ILE A 47 -19.933 8.804 -2.503 1.00 10.00 A

ATOM 419 CG1 ILE A 47 -20.346 8.425 -1.079 1.00 10.00 A

ATOM 420 CG2 ILE A 47 -21.101 9.414 -3.262 1.00 10.00 A

ATOM 421 CD1 ILE A 47 -20.519 9.612 -0.159 1.00 10.00 A

ATOM 422 C ILE A 47 -18.105 7.081 -2.575 1.00 10.00 A

ATOM 423 O ILE A 47 -18.155 6.149 -1.770 1.00 10.00 A

ATOM 424 N LEU A 48 -16.976 7.694 -2.901 1.00 10.00 A

ATOM 425 HN LEU A 48 -16.997 8.437 -3.537 1.00 10.00 A

ATOM 426 CA LEU A 48 -15.702 7.295 -2.323 1.00 10.00 A

ATOM 427 CB LEU A 48 -14.639 7.112 -3.411 1.00 10.00 A

ATOM 428 CG LEU A 48 -14.583 5.737 -4.089 1.00 10.00 A

ATOM 429 CD1 LEU A 48 -14.573 4.619 -3.056 1.00 10.00 A

ATOM 430 CD2 LEU A 48 -15.726 5.559 -5.080 1.00 10.00 A

ATOM 431 C LEU A 48 -15.235 8.311 -1.295 1.00 10.00 A

ATOM 432 O LEU A 48 -15.202 9.514 -1.563 1.00 10.00 A

ATOM 433 N GLU A 49 -14.884 7.822 -0.119 1.00 10.00 A

ATOM 434 HN GLU A 49 -14.939 6.851 0.031 1.00 10.00 A

ATOM 435 CA GLU A 49 -14.423 8.682 0.959 1.00 10.00 A

ATOM 436 CB GLU A 49 -15.415 8.639 2.125 1.00 10.00 A

ATOM 437 CG GLU A 49 -16.861 8.832 1.695 1.00 10.00 A

ATOM 438 CD GLU A 49 -17.818 8.943 2.857 1.00 10.00 A

ATOM 439 OE1 GLU A 49 -18.154 10.079 3.248 1.00 10.00 A

ATOM 440 OE2 GLU A 49 -18.241 7.897 3.390 1.00 10.00 A

ATOM 441 C GLU A 49 -13.023 8.272 1.412 1.00 10.00 A

ATOM 442 O GLU A 49 -12.713 7.078 1.485 1.00 10.00 A

ATOM 443 N PRO A 50 -12.155 9.257 1.696 1.00 10.00 A

ATOM 444 CA PRO A 50 -10.775 9.007 2.132 1.00 10.00 A

ATOM 445 CB PRO A 50 -10.153 10.409 2.209 1.00 10.00 A

ATOM 446 CG PRO A 50 -11.085 11.296 1.455 1.00 10.00 A

ATOM 447 CD PRO A 50 -12.449 10.695 1.612 1.00 10.00 A

ATOM 448 C PRO A 50 -10.708 8.331 3.499 1.00 10.00 A

ATOM 449 O PRO A 50 -11.339 8.780 4.460 1.00 10.00 A

ATOM 450 N LEU A 51 -9.939 7.252 3.583 1.00 10.00 A

ATOM 451 HN LEU A 51 -9.464 6.934 2.783 1.00 10.00 A

ATOM 452 CA LEU A 51 -9.794 6.524 4.831 1.00 10.00 A

ATOM 453 CB LEU A 51 -10.232 5.066 4.663 1.00 10.00 A

ATOM 454 CG LEU A 51 -10.340 4.242 5.948 1.00 10.00 A

ATOM 455 CD1 LEU A 51 -11.343 4.863 6.908 1.00 10.00 A

ATOM 456 CD2 LEU A 51 -10.729 2.809 5.626 1.00 10.00 A

ATOM 457 C LEU A 51 -8.362 6.597 5.356 1.00 10.00 A

ATOM 458 O LEU A 51 -8.121 7.103 6.451 1.00 10.00 A

ATOM 459 N LEU A 52 -7.413 6.101 4.570 1.00 10.00 A

ATOM 460 HN LEU A 52 -7.659 5.728 3.697 1.00 10.00 A

ATOM 461 CA LEU A 52 -6.008 6.108 4.976 1.00 10.00 A

ATOM 462 CB LEU A 52 -5.571 4.711 5.421 1.00 10.00 A

ATOM 463 CG LEU A 52 -6.213 4.175 6.701 1.00 10.00 A

ATOM 464 CD1 LEU A 52 -6.039 2.669 6.792 1.00 10.00 A

ATOM 465 CD2 LEU A 52 -5.614 4.854 7.922 1.00 10.00 A

ATOM 466 C LEU A 52 -5.111 6.594 3.846 1.00 10.00 A

ATOM 467 O LEU A 52 -5.409 6.369 2.672 1.00 10.00 A

ATOM 468 N PHE A 53 -4.025 7.268 4.208 1.00 10.00 A

ATOM 469 HN PHE A 53 -3.858 7.429 5.164 1.00 10.00 A

ATOM 470 CA PHE A 53 -3.072 7.784 3.236 1.00 10.00 A

ATOM 471 CB PHE A 53 -3.007 9.314 3.312 1.00 10.00 A

ATOM 472 CG PHE A 53 -4.254 10.023 2.858 1.00 10.00 A

ATOM 473 CD1 PHE A 53 -5.332 10.184 3.714 1.00 10.00 A

ATOM 474 CD2 PHE A 53 -4.338 10.540 1.578 1.00 10.00 A

ATOM 475 CE1 PHE A 53 -6.472 10.846 3.299 1.00 10.00 A

ATOM 476 CE2 PHE A 53 -5.475 11.203 1.153 1.00 10.00 A

ATOM 477 CZ PHE A 53 -6.542 11.356 2.016 1.00 10.00 A

ATOM 478 C PHE A 53 -1.685 7.217 3.514 1.00 10.00 A

ATOM 479 O PHE A 53 -1.159 7.367 4.619 1.00 10.00 A

ATOM 480 N TRP A 54 -1.100 6.560 2.522 1.00 10.00 A

ATOM 481 HN TRP A 54 -1.582 6.441 1.673 1.00 10.00 A

ATOM 482 CA TRP A 54 0.233 5.988 2.672 1.00 10.00 A

ATOM 483 CB TRP A 54 0.203 4.472 2.465 1.00 10.00 A

ATOM 484 CG TRP A 54 -0.624 3.744 3.475 1.00 10.00 A

ATOM 485 CD1 TRP A 54 -0.447 3.738 4.827 1.00 10.00 A

ATOM 486 CD2 TRP A 54 -1.756 2.907 3.214 1.00 10.00 A

ATOM 487 NE1 TRP A 54 -1.401 2.953 5.425 1.00 10.00 A

ATOM 488 HE1 TRP A 54 -1.483 2.796 6.393 1.00 10.00 A

ATOM 489 CE2 TRP A 54 -2.215 2.429 4.457 1.00 10.00 A

ATOM 490 CE3 TRP A 54 -2.430 2.514 2.052 1.00 10.00 A

ATOM 491 CZ2 TRP A 54 -3.311 1.579 4.571 1.00 10.00 A

ATOM 492 CZ3 TRP A 54 -3.518 1.671 2.169 1.00 10.00 A

ATOM 493 CH2 TRP A 54 -3.948 1.212 3.419 1.00 10.00 A

ATOM 494 C TRP A 54 1.226 6.630 1.707 1.00 10.00 A

ATOM 495 O TRP A 54 1.310 6.239 0.542 1.00 10.00 A

ATOM 496 N PRO A 55 1.981 7.637 2.173 1.00 10.00 A

ATOM 497 CA PRO A 55 2.978 8.330 1.348 1.00 10.00 A

ATOM 498 CB PRO A 55 3.453 9.484 2.243 1.00 10.00 A

ATOM 499 CG PRO A 55 2.444 9.574 3.340 1.00 10.00 A

ATOM 500 CD PRO A 55 1.926 8.182 3.535 1.00 10.00 A

ATOM 501 C PRO A 55 4.148 7.409 1.011 1.00 10.00 A

ATOM 502 O PRO A 55 4.598 6.637 1.859 1.00 10.00 A

ATOM 503 N TYR A 56 4.646 7.501 -0.218 1.00 10.00 A

ATOM 504 HN TYR A 56 4.261 8.154 -0.844 1.00 10.00 A

ATOM 505 CA TYR A 56 5.757 6.655 -0.660 1.00 10.00 A

ATOM 506 CB TYR A 56 6.018 6.801 -2.161 1.00 10.00 A

ATOM 507 CG TYR A 56 4.877 6.323 -3.030 1.00 10.00 A

ATOM 508 CD1 TYR A 56 3.981 5.363 -2.574 1.00 10.00 A

ATOM 509 CD2 TYR A 56 4.689 6.835 -4.306 1.00 10.00 A

ATOM 510 CE1 TYR A 56 2.933 4.930 -3.360 1.00 10.00 A

ATOM 511 CE2 TYR A 56 3.642 6.405 -5.100 1.00 10.00 A

ATOM 512 CZ TYR A 56 2.768 5.455 -4.622 1.00 10.00 A

ATOM 513 OH TYR A 56 1.719 5.034 -5.406 1.00 10.00 A

ATOM 514 HH TYR A 56 1.736 5.511 -6.248 1.00 10.00 A

ATOM 515 C TYR A 56 7.030 6.925 0.133 1.00 10.00 A

ATOM 516 O TYR A 56 7.804 6.012 0.405 1.00 10.00 A

ATOM 517 N THR A 57 7.233 8.177 0.514 1.00 10.00 A

ATOM 518 HN THR A 57 6.588 8.867 0.258 1.00 10.00 A

ATOM 519 CA THR A 57 8.407 8.560 1.286 1.00 10.00 A

ATOM 520 CB THR A 57 8.564 10.088 1.304 1.00 10.00 A

ATOM 521 OG1 THR A 57 7.797 10.646 0.226 1.00 10.00 A

ATOM 522 HG1 THR A 57 7.194 11.311 0.579 1.00 10.00 A

ATOM 523 CG2 THR A 57 10.024 10.475 1.133 1.00 10.00 A

ATOM 524 C THR A 57 8.306 8.041 2.722 1.00 10.00 A

ATOM 525 O THR A 57 9.282 8.050 3.476 1.00 10.00 A

ATOM 526 N LEU A 58 7.115 7.586 3.090 1.00 10.00 A

ATOM 527 HN LEU A 58 6.383 7.591 2.437 1.00 10.00 A

ATOM 528 CA LEU A 58 6.862 7.057 4.423 1.00 10.00 A

ATOM 529 CB LEU A 58 5.634 7.737 5.044 1.00 10.00 A

ATOM 530 CG LEU A 58 5.875 9.057 5.790 1.00 10.00 A

ATOM 531 CD1 LEU A 58 6.348 10.154 4.847 1.00 10.00 A

ATOM 532 CD2 LEU A 58 4.615 9.494 6.519 1.00 10.00 A

ATOM 533 C LEU A 58 6.648 5.547 4.349 1.00 10.00 A

ATOM 534 O LEU A 58 6.164 4.921 5.296 1.00 10.00 A

ATOM 535 N LEU A 59 7.011 4.972 3.210 1.00 10.00 A

ATOM 536 HN LEU A 59 7.398 5.523 2.498 1.00 10.00 A

ATOM 537 CA LEU A 59 6.874 3.542 2.993 1.00 10.00 A

ATOM 538 CB LEU A 59 6.411 3.266 1.561 1.00 10.00 A

ATOM 539 CG LEU A 59 5.810 1.885 1.292 1.00 10.00 A

ATOM 540 CD1 LEU A 59 4.512 1.703 2.070 1.00 10.00 A

ATOM 541 CD2 LEU A 59 5.581 1.687 -0.197 1.00 10.00 A

ATOM 542 C LEU A 59 8.209 2.859 3.256 1.00 10.00 A

ATOM 543 O LEU A 59 9.256 3.509 3.242 1.00 10.00 A

ATOM 544 N ARG A 60 8.173 1.558 3.509 1.00 10.00 A

ATOM 545 HN ARG A 60 7.310 1.093 3.510 1.00 10.00 A

ATOM 546 CA ARG A 60 9.387 0.808 3.785 1.00 10.00 A

ATOM 547 CB ARG A 60 9.335 0.205 5.190 1.00 10.00 A

ATOM 548 CG ARG A 60 9.281 1.233 6.306 1.00 10.00 A

ATOM 549 CD ARG A 60 8.685 0.633 7.565 1.00 10.00 A

ATOM 550 NE ARG A 60 9.097 1.354 8.763 1.00 10.00 A

ATOM 551 HE ARG A 60 8.943 2.336 8.783 1.00 10.00 A

ATOM 552 CZ ARG A 60 9.658 0.782 9.822 1.00 10.00 A

ATOM 553 NH1 ARG A 60 9.851 -0.534 9.855 1.00 10.00 A

ATOM 554 HH11 ARG A 60 9.560 -1.135 9.042 1.00 10.00 A

ATOM 555 HH12 ARG A 60 10.284 -0.978 10.691 1.00 10.00 A

ATOM 556 NH2 ARG A 60 10.012 1.532 10.854 1.00 10.00 A

ATOM 557 HH21 ARG A 60 9.842 2.577 10.831 1.00 10.00 A

ATOM 558 HH22 ARG A 60 10.454 1.101 11.690 1.00 10.00 A

ATOM 559 C ARG A 60 9.633 -0.284 2.746 1.00 10.00 A

ATOM 560 O ARG A 60 10.205 -0.030 1.688 1.00 10.00 A

ATOM 561 N ARG A 61 9.185 -1.493 3.048 1.00 10.00 A

ATOM 562 HN ARG A 61 8.698 -1.627 3.887 1.00 10.00 A

ATOM 563 CA ARG A 61 9.378 -2.628 2.153 1.00 10.00 A

ATOM 564 CB ARG A 61 9.887 -3.838 2.936 1.00 10.00 A

ATOM 565 CG ARG A 61 11.273 -3.638 3.524 1.00 10.00 A

ATOM 566 CD ARG A 61 11.637 -4.741 4.495 1.00 10.00 A

ATOM 567 NE ARG A 61 13.066 -4.734 4.803 1.00 10.00 A

ATOM 568 HE ARG A 61 13.663 -4.324 4.140 1.00 10.00 A

ATOM 569 CZ ARG A 61 13.595 -5.257 5.903 1.00 10.00 A

ATOM 570 NH1 ARG A 61 12.818 -5.834 6.804 1.00 10.00 A

ATOM 571 HH11 ARG A 61 11.788 -5.880 6.657 1.00 10.00 A

ATOM 572 HH12 ARG A 61 13.232 -6.262 7.660 1.00 10.00 A

ATOM 573 NH2 ARG A 61 14.910 -5.210 6.092 1.00 10.00 A

ATOM 574 HH21 ARG A 61 15.528 -4.766 5.376 1.00 10.00 A

ATOM 575 HH22 ARG A 61 15.332 -5.616 6.956 1.00 10.00 A

ATOM 576 C ARG A 61 8.091 -2.973 1.415 1.00 10.00 A

ATOM 577 O ARG A 61 7.011 -2.509 1.787 1.00 10.00 A

ATOM 578 N TYR A 62 8.213 -3.790 0.376 1.00 10.00 A

ATOM 579 HN TYR A 62 9.103 -4.141 0.142 1.00 10.00 A

ATOM 580 CA TYR A 62 7.062 -4.196 -0.420 1.00 10.00 A

ATOM 581 CB TYR A 62 6.693 -3.109 -1.445 1.00 10.00 A

ATOM 582 CG TYR A 62 7.851 -2.610 -2.289 1.00 10.00 A

ATOM 583 CD1 TYR A 62 8.203 -3.256 -3.463 1.00 10.00 A

ATOM 584 CD2 TYR A 62 8.590 -1.493 -1.911 1.00 10.00 A

ATOM 585 CE1 TYR A 62 9.250 -2.809 -4.242 1.00 10.00 A

ATOM 586 CE2 TYR A 62 9.640 -1.037 -2.686 1.00 10.00 A

ATOM 587 CZ TYR A 62 9.968 -1.702 -3.848 1.00 10.00 A

ATOM 588 OH TYR A 62 11.015 -1.261 -4.624 1.00 10.00 A

ATOM 589 HH TYR A 62 10.841 -1.474 -5.558 1.00 10.00 A

ATOM 590 C TYR A 62 7.319 -5.536 -1.111 1.00 10.00 A

ATOM 591 O TYR A 62 8.432 -5.811 -1.559 1.00 10.00 A

ATOM 592 N GLY A 63 6.287 -6.365 -1.189 1.00 10.00 A

ATOM 593 HN GLY A 63 5.417 -6.087 -0.817 1.00 10.00 A

ATOM 594 CA GLY A 63 6.412 -7.667 -1.818 1.00 10.00 A

ATOM 595 C GLY A 63 5.293 -7.926 -2.805 1.00 10.00 A

ATOM 596 O GLY A 63 4.164 -7.474 -2.604 1.00 10.00 A

ATOM 597 N ARG A 64 5.597 -8.664 -3.862 1.00 10.00 A

ATOM 598 HN ARG A 64 6.504 -9.027 -3.946 1.00 10.00 A

ATOM 599 CA ARG A 64 4.613 -8.968 -4.893 1.00 10.00 A

ATOM 600 CB ARG A 64 5.136 -8.523 -6.263 1.00 10.00 A

ATOM 601 CG ARG A 64 4.348 -9.043 -7.456 1.00 10.00 A

ATOM 602 CD ARG A 64 5.150 -10.075 -8.234 1.00 10.00 A

ATOM 603 NE ARG A 64 5.276 -9.726 -9.647 1.00 10.00 A

ATOM 604 HE ARG A 64 4.489 -9.873 -10.220 1.00 10.00 A

ATOM 605 CZ ARG A 64 6.378 -9.226 -10.201 1.00 10.00 A

ATOM 606 NH1 ARG A 64 7.472 -9.034 -9.465 1.00 10.00 A

ATOM 607 HH11 ARG A 64 7.473 -9.276 -8.455 1.00 10.00 A

ATOM 608 HH12 ARG A 64 8.334 -8.634 -9.900 1.00 10.00 A

ATOM 609 NH2 ARG A 64 6.395 -8.934 -11.493 1.00 10.00 A

ATOM 610 HH21 ARG A 64 5.543 -9.100 -12.079 1.00 10.00 A

ATOM 611 HH22 ARG A 64 7.251 -8.525 -11.930 1.00 10.00 A

ATOM 612 C ARG A 64 4.281 -10.454 -4.921 1.00 10.00 A

ATOM 613 O ARG A 64 5.176 -11.299 -4.980 1.00 10.00 A

ATOM 614 N ASP A 65 2.994 -10.760 -4.876 1.00 10.00 A

ATOM 615 HN ASP A 65 2.331 -10.038 -4.808 1.00 10.00 A

ATOM 616 CA ASP A 65 2.524 -12.136 -4.927 1.00 10.00 A

ATOM 617 CB ASP A 65 1.835 -12.521 -3.612 1.00 10.00 A

ATOM 618 CG ASP A 65 1.260 -13.924 -3.626 1.00 10.00 A

ATOM 619 OD1 ASP A 65 2.044 -14.891 -3.637 1.00 10.00 A

ATOM 620 OD2 ASP A 65 0.020 -14.059 -3.649 1.00 10.00 A

ATOM 621 C ASP A 65 1.575 -12.291 -6.115 1.00 10.00 A

ATOM 622 O ASP A 65 1.160 -11.298 -6.717 1.00 10.00 A

ATOM 623 N LYS A 66 1.250 -13.523 -6.465 1.00 10.00 A

ATOM 624 HN LYS A 66 1.611 -14.277 -5.950 1.00 10.00 A

ATOM 625 CA LYS A 66 0.367 -13.790 -7.588 1.00 10.00 A

ATOM 626 CB LYS A 66 0.609 -15.209 -8.109 1.00 10.00 A

ATOM 627 CG LYS A 66 -0.083 -15.528 -9.425 1.00 10.00 A

ATOM 628 CD LYS A 66 0.297 -16.915 -9.919 1.00 10.00 A

ATOM 629 CE LYS A 66 -0.393 -17.248 -11.232 1.00 10.00 A

ATOM 630 NZ LYS A 66 0.040 -18.565 -11.772 1.00 10.00 A

ATOM 631 HZ1 LYS A 66 -0.100 -19.308 -11.058 1.00 10.00 A

ATOM 632 HZ2 LYS A 66 1.046 -18.535 -12.035 1.00 10.00 A

ATOM 633 HZ3 LYS A 66 -0.519 -18.804 -12.617 1.00 10.00 A

ATOM 634 C LYS A 66 -1.100 -13.612 -7.203 1.00 10.00 A

ATOM 635 O LYS A 66 -1.946 -13.337 -8.056 1.00 10.00 A

ATOM 636 N VAL A 67 -1.404 -13.759 -5.922 1.00 10.00 A

ATOM 637 HN VAL A 67 -0.689 -13.959 -5.269 1.00 10.00 A

ATOM 638 CA VAL A 67 -2.778 -13.633 -5.455 1.00 10.00 A

ATOM 639 CB VAL A 67 -3.263 -14.917 -4.734 1.00 10.00 A

ATOM 640 CG1 VAL A 67 -4.784 -14.978 -4.696 1.00 10.00 A

ATOM 641 CG2 VAL A 67 -2.693 -16.166 -5.392 1.00 10.00 A

ATOM 642 C VAL A 67 -2.958 -12.431 -4.523 1.00 10.00 A

ATOM 643 O VAL A 67 -4.085 -12.030 -4.225 1.00 10.00 A

ATOM 644 N MET A 68 -1.860 -11.845 -4.060 1.00 10.00 A

ATOM 645 HN MET A 68 -0.976 -12.187 -4.323 1.00 10.00 A

ATOM 646 CA MET A 68 -1.953 -10.702 -3.157 1.00 10.00 A

ATOM 647 CB MET A 68 -2.095 -11.170 -1.701 1.00 10.00 A

ATOM 648 CG MET A 68 -0.885 -11.907 -1.143 1.00 10.00 A

ATOM 649 SD MET A 68 0.318 -10.806 -0.368 1.00 10.00 A

ATOM 650 CE MET A 68 1.599 -11.972 0.090 1.00 10.00 A

ATOM 651 C MET A 68 -0.779 -9.735 -3.292 1.00 10.00 A

ATOM 652 O MET A 68 0.172 -9.978 -4.034 1.00 10.00 A

ATOM 653 N PHE A 69 -0.878 -8.626 -2.575 1.00 10.00 A

ATOM 654 HN PHE A 69 -1.688 -8.483 -2.034 1.00 10.00 A

ATOM 655 CA PHE A 69 0.156 -7.608 -2.553 1.00 10.00 A

ATOM 656 CB PHE A 69 -0.246 -6.416 -3.425 1.00 10.00 A

ATOM 657 CG PHE A 69 0.792 -5.329 -3.494 1.00 10.00 A

ATOM 658 CD1 PHE A 69 1.971 -5.524 -4.196 1.00 10.00 A

ATOM 659 CD2 PHE A 69 0.587 -4.112 -2.863 1.00 10.00 A

ATOM 660 CE1 PHE A 69 2.926 -4.527 -4.264 1.00 10.00 A

ATOM 661 CE2 PHE A 69 1.539 -3.111 -2.928 1.00 10.00 A

ATOM 662 CZ PHE A 69 2.709 -3.320 -3.631 1.00 10.00 A

ATOM 663 C PHE A 69 0.356 -7.160 -1.111 1.00 10.00 A

ATOM 664 O PHE A 69 -0.618 -6.957 -0.382 1.00 10.00 A

ATOM 665 N SER A 70 1.605 -7.021 -0.696 1.00 10.00 A

ATOM 666 HN SER A 70 2.346 -7.180 -1.323 1.00 10.00 A

ATOM 667 CA SER A 70 1.900 -6.616 0.669 1.00 10.00 A

ATOM 668 CB SER A 70 2.322 -7.825 1.508 1.00 10.00 A

ATOM 669 OG SER A 70 1.252 -8.739 1.673 1.00 10.00 A

ATOM 670 HG SER A 70 1.042 -9.146 0.820 1.00 10.00 A

ATOM 671 C SER A 70 2.984 -5.549 0.726 1.00 10.00 A

ATOM 672 O SER A 70 3.937 -5.570 -0.050 1.00 10.00 A

ATOM 673 N PHE A 71 2.824 -4.617 1.652 1.00 10.00 A

ATOM 674 HN PHE A 71 2.027 -4.648 2.226 1.00 10.00 A

ATOM 675 CA PHE A 71 3.789 -3.549 1.847 1.00 10.00 A

ATOM 676 CB PHE A 71 3.422 -2.291 1.043 1.00 10.00 A

ATOM 677 CG PHE A 71 2.080 -1.688 1.365 1.00 10.00 A

ATOM 678 CD1 PHE A 71 1.947 -0.759 2.387 1.00 10.00 A

ATOM 679 CD2 PHE A 71 0.955 -2.039 0.639 1.00 10.00 A

ATOM 680 CE1 PHE A 71 0.720 -0.192 2.677 1.00 10.00 A

ATOM 681 CE2 PHE A 71 -0.275 -1.478 0.923 1.00 10.00 A

ATOM 682 CZ PHE A 71 -0.394 -0.553 1.943 1.00 10.00 A

ATOM 683 C PHE A 71 3.930 -3.238 3.329 1.00 10.00 A

ATOM 684 O PHE A 71 3.001 -3.462 4.106 1.00 10.00 A

ATOM 685 N GLU A 72 5.089 -2.742 3.719 1.00 10.00 A

ATOM 686 HN GLU A 72 5.794 -2.583 3.047 1.00 10.00 A

ATOM 687 CA GLU A 72 5.348 -2.413 5.109 1.00 10.00 A

ATOM 688 CB GLU A 72 6.767 -2.842 5.504 1.00 10.00 A

ATOM 689 CG GLU A 72 7.045 -2.783 7.000 1.00 10.00 A

ATOM 690 CD GLU A 72 8.456 -3.210 7.351 1.00 10.00 A

ATOM 691 OE1 GLU A 72 8.804 -4.383 7.108 1.00 10.00 A

ATOM 692 OE2 GLU A 72 9.221 -2.374 7.880 1.00 10.00 A

ATOM 693 C GLU A 72 5.154 -0.922 5.352 1.00 10.00 A

ATOM 694 O GLU A 72 5.938 -0.096 4.872 1.00 10.00 A

ATOM 695 N ALA A 73 4.099 -0.585 6.081 1.00 10.00 A

ATOM 696 HN ALA A 73 3.511 -1.291 6.428 1.00 10.00 A

ATOM 697 CA ALA A 73 3.801 0.799 6.399 1.00 10.00 A

ATOM 698 CB ALA A 73 2.326 0.970 6.730 1.00 10.00 A

ATOM 699 C ALA A 73 4.674 1.259 7.558 1.00 10.00 A

ATOM 700 O ALA A 73 4.831 0.544 8.553 1.00 10.00 A

ATOM 701 N GLY A 74 5.258 2.438 7.411 1.00 10.00 A

ATOM 702 HN GLY A 74 5.096 2.955 6.598 1.00 10.00 A

ATOM 703 CA GLY A 74 6.123 2.970 8.438 1.00 10.00 A

ATOM 704 C GLY A 74 5.371 3.482 9.646 1.00 10.00 A

ATOM 705 O GLY A 74 4.153 3.664 9.604 1.00 10.00 A

ATOM 706 N ARG A 75 6.110 3.733 10.714 1.00 10.00 A

ATOM 707 HN ARG A 75 7.095 3.605 10.650 1.00 10.00 A

ATOM 708 CA ARG A 75 5.541 4.236 11.958 1.00 10.00 A

ATOM 709 CB ARG A 75 6.563 4.073 13.095 1.00 10.00 A

ATOM 710 CG ARG A 75 6.279 4.869 14.365 1.00 10.00 A

ATOM 711 CD ARG A 75 7.107 6.147 14.413 1.00 10.00 A

ATOM 712 NE ARG A 75 8.340 6.026 13.635 1.00 10.00 A

ATOM 713 HE ARG A 75 8.258 5.676 12.705 1.00 10.00 A

ATOM 714 CZ ARG A 75 9.554 6.326 14.086 1.00 10.00 A

ATOM 715 NH1 ARG A 75 9.720 6.842 15.298 1.00 10.00 A

ATOM 716 HH11 ARG A 75 8.897 7.021 15.914 1.00 10.00 A

ATOM 717 HH12 ARG A 75 10.680 7.061 15.643 1.00 10.00 A

ATOM 718 NH2 ARG A 75 10.604 6.115 13.314 1.00 10.00 A

ATOM 719 HH21 ARG A 75 10.472 5.706 12.348 1.00 10.00 A

ATOM 720 HH22 ARG A 75 11.561 6.343 13.651 1.00 10.00 A

ATOM 721 C ARG A 75 5.147 5.694 11.774 1.00 10.00 A

ATOM 722 O ARG A 75 4.256 6.215 12.449 1.00 10.00 A

ATOM 723 N ARG A 76 5.836 6.341 10.845 1.00 10.00 A

ATOM 724 HN ARG A 76 6.568 5.866 10.383 1.00 10.00 A

ATOM 725 CA ARG A 76 5.583 7.731 10.510 1.00 10.00 A

ATOM 726 CB ARG A 76 6.704 8.262 9.613 1.00 10.00 A

ATOM 727 CG ARG A 76 6.843 9.773 9.606 1.00 10.00 A

ATOM 728 CD ARG A 76 8.064 10.209 8.819 1.00 10.00 A

ATOM 729 NE ARG A 76 8.228 11.658 8.841 1.00 10.00 A

ATOM 730 HE ARG A 76 7.454 12.194 9.136 1.00 10.00 A

ATOM 731 CZ ARG A 76 9.349 12.290 8.500 1.00 10.00 A

ATOM 732 NH1 ARG A 76 10.392 11.603 8.058 1.00 10.00 A

ATOM 733 HH11 ARG A 76 10.341 10.566 7.969 1.00 10.00 A

ATOM 734 HH12 ARG A 76 11.271 12.098 7.797 1.00 10.00 A

ATOM 735 NH2 ARG A 76 9.419 13.612 8.581 1.00 10.00 A

ATOM 736 HH21 ARG A 76 8.595 14.165 8.908 1.00 10.00 A

ATOM 737 HH22 ARG A 76 10.300 14.106 8.324 1.00 10.00 A

ATOM 738 C ARG A 76 4.231 7.866 9.811 1.00 10.00 A

ATOM 739 O ARG A 76 3.654 8.953 9.755 1.00 10.00 A

ATOM 740 N CYS A 77 3.726 6.754 9.290 1.00 10.00 A

ATOM 741 HN CYS A 77 4.225 5.914 9.380 1.00 10.00 A

ATOM 742 CA CYS A 77 2.439 6.741 8.608 1.00 10.00 A

ATOM 743 CB CYS A 77 2.280 5.467 7.771 1.00 10.00 A

ATOM 744 SG CYS A 77 3.147 5.498 6.184 1.00 10.00 A

ATOM 745 HG CYS A 77 4.418 5.186 6.401 1.00 10.00 A

ATOM 746 C CYS A 77 1.298 6.865 9.616 1.00 10.00 A

ATOM 747 O CYS A 77 1.393 6.358 10.735 1.00 10.00 A

ATOM 748 N PRO A 78 0.201 7.536 9.223 1.00 10.00 A

ATOM 749 CA PRO A 78 -0.966 7.751 10.094 1.00 10.00 A

ATOM 750 CB PRO A 78 -1.980 8.423 9.165 1.00 10.00 A

ATOM 751 CG PRO A 78 -1.153 9.065 8.106 1.00 10.00 A

ATOM 752 CD PRO A 78 0.018 8.154 7.896 1.00 10.00 A

ATOM 753 C PRO A 78 -1.549 6.450 10.649 1.00 10.00 A

ATOM 754 O PRO A 78 -2.033 6.409 11.783 1.00 10.00 A

ATOM 755 N SER A 79 -1.490 5.387 9.853 1.00 10.00 A

ATOM 756 HN SER A 79 -1.086 5.477 8.965 1.00 10.00 A

ATOM 757 CA SER A 79 -2.020 4.091 10.258 1.00 10.00 A

ATOM 758 CB SER A 79 -2.224 3.220 9.021 1.00 10.00 A

ATOM 759 OG SER A 79 -1.095 3.293 8.166 1.00 10.00 A

ATOM 760 HG SER A 79 -0.348 2.849 8.586 1.00 10.00 A

ATOM 761 C SER A 79 -1.097 3.381 11.251 1.00 10.00 A

ATOM 762 O SER A 79 -1.448 2.336 11.806 1.00 10.00 A

ATOM 763 N GLY A 80 0.077 3.955 11.476 1.00 10.00 A

ATOM 764 HN GLY A 80 0.298 4.796 11.019 1.00 10.00 A

ATOM 765 CA GLY A 80 1.032 3.361 12.386 1.00 10.00 A

ATOM 766 C GLY A 80 1.887 2.324 11.686 1.00 10.00 A

ATOM 767 O GLY A 80 1.633 1.996 10.527 1.00 10.00 A

ATOM 768 N PRO A 81 2.896 1.773 12.371 1.00 10.00 A

ATOM 769 CA PRO A 81 3.786 0.768 11.785 1.00 10.00 A

ATOM 770 CB PRO A 81 4.937 0.697 12.789 1.00 10.00 A

ATOM 771 CG PRO A 81 4.320 1.074 14.090 1.00 10.00 A

ATOM 772 CD PRO A 81 3.244 2.076 13.770 1.00 10.00 A

ATOM 773 C PRO A 81 3.109 -0.595 11.663 1.00 10.00 A

ATOM 774 O PRO A 81 2.355 -1.013 12.550 1.00 10.00 A

ATOM 775 N GLY A 82 3.352 -1.270 10.556 1.00 10.00 A

ATOM 776 HN GLY A 82 3.927 -0.874 9.860 1.00 10.00 A

ATOM 777 CA GLY A 82 2.764 -2.575 10.354 1.00 10.00 A

ATOM 778 C GLY A 82 2.675 -2.954 8.893 1.00 10.00 A

ATOM 779 O GLY A 82 2.618 -2.088 8.021 1.00 10.00 A

ATOM 780 N THR A 83 2.678 -4.246 8.627 1.00 10.00 A

ATOM 781 HN THR A 83 2.754 -4.889 9.363 1.00 10.00 A

ATOM 782 CA THR A 83 2.589 -4.749 7.271 1.00 10.00 A

ATOM 783 CB THR A 83 3.201 -6.159 7.189 1.00 10.00 A

ATOM 784 OG1 THR A 83 3.936 -6.425 8.397 1.00 10.00 A

ATOM 785 HG1 THR A 83 4.876 -6.262 8.244 1.00 10.00 A

ATOM 786 CG2 THR A 83 4.138 -6.266 5.994 1.00 10.00 A

ATOM 787 C THR A 83 1.131 -4.806 6.820 1.00 10.00 A

ATOM 788 O THR A 83 0.256 -5.236 7.574 1.00 10.00 A

ATOM 789 N PHE A 84 0.873 -4.350 5.604 1.00 10.00 A

ATOM 790 HN PHE A 84 1.610 -3.997 5.055 1.00 10.00 A

ATOM 791 CA PHE A 84 -0.473 -4.364 5.053 1.00 10.00 A

ATOM 792 CB PHE A 84 -0.878 -2.970 4.572 1.00 10.00 A

ATOM 793 CG PHE A 84 -1.447 -2.087 5.647 1.00 10.00 A

ATOM 794 CD1 PHE A 84 -0.671 -1.101 6.235 1.00 10.00 A

ATOM 795 CD2 PHE A 84 -2.760 -2.236 6.063 1.00 10.00 A

ATOM 796 CE1 PHE A 84 -1.191 -0.281 7.219 1.00 10.00 A

ATOM 797 CE2 PHE A 84 -3.287 -1.421 7.046 1.00 10.00 A

ATOM 798 CZ PHE A 84 -2.504 -0.441 7.624 1.00 10.00 A

ATOM 799 C PHE A 84 -0.562 -5.356 3.905 1.00 10.00 A

ATOM 800 O PHE A 84 0.222 -5.288 2.955 1.00 10.00 A

ATOM 801 N THR A 85 -1.502 -6.281 4.006 1.00 10.00 A

ATOM 802 HN THR A 85 -2.084 -6.288 4.795 1.00 10.00 A

ATOM 803 CA THR A 85 -1.696 -7.288 2.979 1.00 10.00 A

ATOM 804 CB THR A 85 -1.516 -8.712 3.546 1.00 10.00 A

ATOM 805 OG1 THR A 85 -0.157 -8.877 3.973 1.00 10.00 A

ATOM 806 HG1 THR A 85 0.435 -8.684 3.227 1.00 10.00 A

ATOM 807 CG2 THR A 85 -1.836 -9.762 2.490 1.00 10.00 A

ATOM 808 C THR A 85 -3.071 -7.156 2.328 1.00 10.00 A

ATOM 809 O THR A 85 -4.106 -7.230 3.000 1.00 10.00 A

ATOM 810 N PHE A 86 -3.071 -6.943 1.022 1.00 10.00 A

ATOM 811 HN PHE A 86 -2.208 -6.873 0.549 1.00 10.00 A

ATOM 812 CA PHE A 86 -4.301 -6.819 0.263 1.00 10.00 A

ATOM 813 CB PHE A 86 -4.415 -5.427 -0.364 1.00 10.00 A

ATOM 814 CG PHE A 86 -4.560 -4.311 0.631 1.00 10.00 A

ATOM 815 CD1 PHE A 86 -3.462 -3.557 1.013 1.00 10.00 A

ATOM 816 CD2 PHE A 86 -5.795 -4.012 1.183 1.00 10.00 A

ATOM 817 CE1 PHE A 86 -3.592 -2.529 1.927 1.00 10.00 A

ATOM 818 CE2 PHE A 86 -5.931 -2.986 2.097 1.00 10.00 A

ATOM 819 CZ PHE A 86 -4.829 -2.243 2.469 1.00 10.00 A

ATOM 820 C PHE A 86 -4.316 -7.871 -0.830 1.00 10.00 A

ATOM 821 O PHE A 86 -3.424 -7.901 -1.674 1.00 10.00 A

ATOM 822 N GLN A 87 -5.300 -8.752 -0.807 1.00 10.00 A

ATOM 823 HN GLN A 87 -5.988 -8.696 -0.105 1.00 10.00 A

ATOM 824 CA GLN A 87 -5.382 -9.791 -1.819 1.00 10.00 A

ATOM 825 CB GLN A 87 -6.080 -11.057 -1.302 1.00 10.00 A

ATOM 826 CG GLN A 87 -7.593 -10.973 -1.180 1.00 10.00 A

ATOM 827 CD GLN A 87 -8.310 -11.455 -2.428 1.00 10.00 A

ATOM 828 OE1 GLN A 87 -9.410 -11.000 -2.739 1.00 10.00 A

ATOM 829 NE2 GLN A 87 -7.692 -12.377 -3.153 1.00 10.00 A

ATOM 830 HE21 GLN A 87 -6.816 -12.696 -2.851 1.00 10.00 A

ATOM 831 HE22 GLN A 87 -8.138 -12.703 -3.964 1.00 10.00 A

ATOM 832 C GLN A 87 -6.006 -9.255 -3.103 1.00 10.00 A

ATOM 833 O GLN A 87 -7.055 -8.607 -3.075 1.00 10.00 A

ATOM 834 N THR A 88 -5.338 -9.507 -4.217 1.00 10.00 A

ATOM 835 HN THR A 88 -4.509 -10.031 -4.171 1.00 10.00 A

ATOM 836 CA THR A 88 -5.798 -9.048 -5.514 1.00 10.00 A

ATOM 837 CB THR A 88 -5.828 -7.501 -5.592 1.00 10.00 A

ATOM 838 OG1 THR A 88 -6.212 -7.084 -6.906 1.00 10.00 A

ATOM 839 HG1 THR A 88 -7.171 -7.158 -6.994 1.00 10.00 A

ATOM 840 CG2 THR A 88 -4.472 -6.900 -5.244 1.00 10.00 A

ATOM 841 C THR A 88 -4.897 -9.598 -6.615 1.00 10.00 A

ATOM 842 O THR A 88 -3.681 -9.707 -6.441 1.00 10.00 A

ATOM 843 N SER A 89 -5.495 -9.949 -7.743 1.00 10.00 A

ATOM 844 HN SER A 89 -6.471 -9.851 -7.821 1.00 10.00 A

ATOM 845 CA SER A 89 -4.746 -10.482 -8.870 1.00 10.00 A

ATOM 846 CB SER A 89 -5.705 -11.127 -9.871 1.00 10.00 A

ATOM 847 OG SER A 89 -6.775 -11.773 -9.196 1.00 10.00 A

ATOM 848 HG SER A 89 -6.442 -12.561 -8.747 1.00 10.00 A

ATOM 849 C SER A 89 -3.927 -9.383 -9.547 1.00 10.00 A

ATOM 850 O SER A 89 -3.050 -9.657 -10.367 1.00 10.00 A

ATOM 851 N GLN A 90 -4.208 -8.134 -9.186 1.00 10.00 A

ATOM 852 HN GLN A 90 -4.910 -7.973 -8.513 1.00 10.00 A

ATOM 853 CA GLN A 90 -3.500 -6.997 -9.756 1.00 10.00 A

ATOM 854 CB GLN A 90 -4.373 -5.741 -9.731 1.00 10.00 A

ATOM 855 CG GLN A 90 -5.559 -5.769 -10.678 1.00 10.00 A

ATOM 856 CD GLN A 90 -6.832 -6.243 -10.007 1.00 10.00 A

ATOM 857 OE1 GLN A 90 -7.554 -5.456 -9.395 1.00 10.00 A

ATOM 858 NE2 GLN A 90 -7.124 -7.529 -10.133 1.00 10.00 A

ATOM 859 HE21 GLN A 90 -6.508 -8.096 -10.648 1.00 10.00 A

ATOM 860 HE22 GLN A 90 -7.946 -7.862 -9.713 1.00 10.00 A

ATOM 861 C GLN A 90 -2.203 -6.724 -9.006 1.00 10.00 A

ATOM 862 O GLN A 90 -1.446 -5.830 -9.385 1.00 10.00 A

ATOM 863 N GLY A 91 -1.948 -7.501 -7.953 1.00 10.00 A

ATOM 864 HN GLY A 91 -2.587 -8.208 -7.717 1.00 10.00 A

ATOM 865 CA GLY A 91 -0.745 -7.322 -7.150 1.00 10.00 A

ATOM 866 C GLY A 91 0.533 -7.267 -7.970 1.00 10.00 A

ATOM 867 O GLY A 91 1.429 -6.474 -7.681 1.00 10.00 A

ATOM 868 N ASN A 92 0.609 -8.105 -8.996 1.00 10.00 A

ATOM 869 HN ASN A 92 -0.139 -8.715 -9.164 1.00 10.00 A

ATOM 870 CA ASN A 92 1.778 -8.157 -9.871 1.00 10.00 A

ATOM 871 CB ASN A 92 1.653 -9.321 -10.861 1.00 10.00 A

ATOM 872 CG ASN A 92 2.504 -9.143 -12.107 1.00 10.00 A

ATOM 873 OD1 ASN A 92 3.711 -9.378 -12.092 1.00 10.00 A

ATOM 874 ND2 ASN A 92 1.880 -8.734 -13.198 1.00 10.00 A

ATOM 875 HD21 ASN A 92 0.912 -8.571 -13.148 1.00 10.00 A

ATOM 876 HD22 ASN A 92 2.409 -8.612 -14.013 1.00 10.00 A

ATOM 877 C ASN A 92 1.993 -6.841 -10.619 1.00 10.00 A

ATOM 878 O ASN A 92 3.126 -6.382 -10.774 1.00 10.00 A

ATOM 879 N ASP A 93 0.909 -6.236 -11.081 1.00 10.00 A

ATOM 880 HN ASP A 93 0.028 -6.640 -10.920 1.00 10.00 A

ATOM 881 CA ASP A 93 0.996 -4.981 -11.821 1.00 10.00 A

ATOM 882 CB ASP A 93 -0.225 -4.792 -12.720 1.00 10.00 A

ATOM 883 CG ASP A 93 0.089 -3.990 -13.968 1.00 10.00 A

ATOM 884 OD1 ASP A 93 0.726 -4.539 -14.891 1.00 10.00 A

ATOM 885 OD2 ASP A 93 -0.314 -2.813 -14.046 1.00 10.00 A

ATOM 886 C ASP A 93 1.153 -3.794 -10.875 1.00 10.00 A

ATOM 887 O ASP A 93 1.921 -2.870 -11.147 1.00 10.00 A

ATOM 888 N ILE A 94 0.433 -3.836 -9.755 1.00 10.00 A

ATOM 889 HN ILE A 94 -0.163 -4.604 -9.601 1.00 10.00 A

ATOM 890 CA ILE A 94 0.493 -2.773 -8.751 1.00 10.00 A

ATOM 891 CB ILE A 94 -0.464 -3.048 -7.566 1.00 10.00 A

ATOM 892 CG1 ILE A 94 -1.918 -3.000 -8.037 1.00 10.00 A

ATOM 893 CG2 ILE A 94 -0.241 -2.049 -6.435 1.00 10.00 A

ATOM 894 CD1 ILE A 94 -2.920 -3.427 -6.988 1.00 10.00 A

ATOM 895 C ILE A 94 1.919 -2.613 -8.233 1.00 10.00 A

ATOM 896 O ILE A 94 2.394 -1.497 -8.022 1.00 10.00 A

ATOM 897 N PHE A 95 2.594 -3.742 -8.048 1.00 10.00 A

ATOM 898 HN PHE A 95 2.146 -4.596 -8.235 1.00 10.00 A

ATOM 899 CA PHE A 95 3.972 -3.759 -7.571 1.00 10.00 A

ATOM 900 CB PHE A 95 4.489 -5.203 -7.569 1.00 10.00 A

ATOM 901 CG PHE A 95 5.970 -5.357 -7.359 1.00 10.00 A

ATOM 902 CD1 PHE A 95 6.517 -5.282 -6.090 1.00 10.00 A

ATOM 903 CD2 PHE A 95 6.812 -5.591 -8.434 1.00 10.00 A

ATOM 904 CE1 PHE A 95 7.875 -5.441 -5.895 1.00 10.00 A

ATOM 905 CE2 PHE A 95 8.171 -5.747 -8.245 1.00 10.00 A

ATOM 906 CZ PHE A 95 8.704 -5.670 -6.974 1.00 10.00 A

ATOM 907 C PHE A 95 4.861 -2.883 -8.449 1.00 10.00 A

ATOM 908 O PHE A 95 5.643 -2.076 -7.951 1.00 10.00 A

ATOM 909 N GLN A 96 4.712 -3.038 -9.756 1.00 10.00 A

ATOM 910 HN GLN A 96 4.044 -3.673 -10.084 1.00 10.00 A

ATOM 911 CA GLN A 96 5.500 -2.282 -10.715 1.00 10.00 A

ATOM 912 CB GLN A 96 5.325 -2.866 -12.118 1.00 10.00 A

ATOM 913 CG GLN A 96 5.438 -4.382 -12.171 1.00 10.00 A

ATOM 914 CD GLN A 96 5.030 -4.948 -13.515 1.00 10.00 A

ATOM 915 OE1 GLN A 96 5.255 -4.336 -14.560 1.00 10.00 A

ATOM 916 NE2 GLN A 96 4.411 -6.112 -13.496 1.00 10.00 A

ATOM 917 HE21 GLN A 96 4.254 -6.537 -12.625 1.00 10.00 A

ATOM 918 HE22 GLN A 96 4.131 -6.504 -14.352 1.00 10.00 A

ATOM 919 C GLN A 96 5.102 -0.812 -10.706 1.00 10.00 A

ATOM 920 O GLN A 96 5.958 0.066 -10.756 1.00 10.00 A

ATOM 921 N ALA A 97 3.801 -0.557 -10.630 1.00 10.00 A

ATOM 922 HN ALA A 97 3.169 -1.310 -10.583 1.00 10.00 A

ATOM 923 CA ALA A 97 3.282 0.806 -10.618 1.00 10.00 A

ATOM 924 CB ALA A 97 1.761 0.795 -10.623 1.00 10.00 A

ATOM 925 C ALA A 97 3.807 1.592 -9.421 1.00 10.00 A

ATOM 926 O ALA A 97 4.324 2.701 -9.576 1.00 10.00 A

ATOM 927 N VAL A 98 3.680 1.009 -8.229 1.00 10.00 A

ATOM 928 HN VAL A 98 3.261 0.119 -8.173 1.00 10.00 A

ATOM 929 CA VAL A 98 4.143 1.658 -7.005 1.00 10.00 A

ATOM 930 CB VAL A 98 3.798 0.826 -5.748 1.00 10.00 A

ATOM 931 CG1 VAL A 98 4.367 1.475 -4.492 1.00 10.00 A

ATOM 932 CG2 VAL A 98 2.293 0.652 -5.620 1.00 10.00 A

ATOM 933 C VAL A 98 5.646 1.905 -7.061 1.00 10.00 A

ATOM 934 O VAL A 98 6.111 3.019 -6.815 1.00 10.00 A

ATOM 935 N GLU A 99 6.396 0.868 -7.408 1.00 10.00 A

ATOM 936 HN GLU A 99 5.962 0.007 -7.607 1.00 10.00 A

ATOM 937 CA GLU A 99 7.846 0.969 -7.500 1.00 10.00 A

ATOM 938 CB GLU A 99 8.452 -0.385 -7.854 1.00 10.00 A

ATOM 939 CG GLU A 99 9.948 -0.341 -8.092 1.00 10.00 A

ATOM 940 CD GLU A 99 10.559 -1.714 -8.179 1.00 10.00 A

ATOM 941 OE1 GLU A 99 10.584 -2.293 -9.285 1.00 10.00 A

ATOM 942 OE2 GLU A 99 11.038 -2.217 -7.142 1.00 10.00 A

ATOM 943 C GLU A 99 8.262 2.018 -8.524 1.00 10.00 A

ATOM 944 O GLU A 99 9.171 2.812 -8.275 1.00 10.00 A

ATOM 945 N ALA A 100 7.583 2.024 -9.666 1.00 10.00 A

ATOM 946 HN ALA A 100 6.866 1.363 -9.799 1.00 10.00 A

ATOM 947 CA ALA A 100 7.873 2.974 -10.730 1.00 10.00 A

ATOM 948 CB ALA A 100 6.926 2.771 -11.903 1.00 10.00 A

ATOM 949 C ALA A 100 7.784 4.402 -10.218 1.00 10.00 A

ATOM 950 O ALA A 100 8.649 5.230 -10.504 1.00 10.00 A

ATOM 951 N ALA A 101 6.738 4.683 -9.448 1.00 10.00 A

ATOM 952 HN ALA A 101 6.086 3.975 -9.251 1.00 10.00 A

ATOM 953 CA ALA A 101 6.537 6.011 -8.886 1.00 10.00 A

ATOM 954 CB ALA A 101 5.181 6.104 -8.209 1.00 10.00 A

ATOM 955 C ALA A 101 7.655 6.362 -7.910 1.00 10.00 A

ATOM 956 O ALA A 101 8.183 7.476 -7.930 1.00 10.00 A

ATOM 957 N ILE A 102 8.025 5.401 -7.073 1.00 10.00 A

ATOM 958 HN ILE A 102 7.563 4.534 -7.106 1.00 10.00 A

ATOM 959 CA ILE A 102 9.088 5.602 -6.095 1.00 10.00 A

ATOM 960 CB ILE A 102 9.249 4.374 -5.174 1.00 10.00 A

ATOM 961 CG1 ILE A 102 7.944 4.102 -4.421 1.00 10.00 A

ATOM 962 CG2 ILE A 102 10.396 4.582 -4.194 1.00 10.00 A

ATOM 963 CD1 ILE A 102 7.940 2.799 -3.653 1.00 10.00 A

ATOM 964 C ILE A 102 10.416 5.898 -6.794 1.00 10.00 A

ATOM 965 O ILE A 102 11.136 6.827 -6.417 1.00 10.00 A

ATOM 966 N GLN A 103 10.726 5.118 -7.827 1.00 10.00 A

ATOM 967 HN GLN A 103 10.104 4.398 -8.089 1.00 10.00 A

ATOM 968 CA GLN A 103 11.962 5.299 -8.580 1.00 10.00 A

ATOM 969 CB GLN A 103 12.161 4.171 -9.597 1.00 10.00 A

ATOM 970 CG GLN A 103 12.289 2.786 -8.980 1.00 10.00 A

ATOM 971 CD GLN A 103 12.770 1.746 -9.973 1.00 10.00 A

ATOM 972 OE1 GLN A 103 12.560 1.873 -11.180 1.00 10.00 A

ATOM 973 NE2 GLN A 103 13.416 0.708 -9.472 1.00 10.00 A

ATOM 974 HE21 GLN A 103 13.549 0.668 -8.501 1.00 10.00 A

ATOM 975 HE22 GLN A 103 13.735 0.016 -10.088 1.00 10.00 A

ATOM 976 C GLN A 103 11.982 6.652 -9.285 1.00 10.00 A

ATOM 977 O GLN A 103 13.041 7.237 -9.485 1.00 10.00 A

ATOM 978 N GLN A 104 10.803 7.141 -9.655 1.00 10.00 A

ATOM 979 HN GLN A 104 9.991 6.616 -9.471 1.00 10.00 A

ATOM 980 CA GLN A 104 10.679 8.430 -10.328 1.00 10.00 A

ATOM 981 CB GLN A 104 9.349 8.518 -11.080 1.00 10.00 A

ATOM 982 CG GLN A 104 9.346 7.810 -12.424 1.00 10.00 A

ATOM 983 CD GLN A 104 7.954 7.652 -13.000 1.00 10.00 A

ATOM 984 OE1 GLN A 104 7.448 8.532 -13.697 1.00 10.00 A

ATOM 985 NE2 GLN A 104 7.327 6.526 -12.713 1.00 10.00 A

ATOM 986 HE21 GLN A 104 7.792 5.870 -12.151 1.00 10.00 A

ATOM 987 HE22 GLN A 104 6.427 6.387 -13.079 1.00 10.00 A

ATOM 988 C GLN A 104 10.788 9.576 -9.327 1.00 10.00 A

ATOM 989 O GLN A 104 11.307 10.650 -9.646 1.00 10.00 A

ATOM 990 N GLN A 105 10.296 9.337 -8.118 1.00 10.00 A

ATOM 991 HN GLN A 105 9.886 8.462 -7.936 1.00 10.00 A

ATOM 992 CA GLN A 105 10.325 10.334 -7.058 1.00 10.00 A

ATOM 993 CB GLN A 105 9.414 9.910 -5.900 1.00 10.00 A

ATOM 994 CG GLN A 105 9.467 10.839 -4.696 1.00 10.00 A

ATOM 995 CD GLN A 105 8.606 10.371 -3.537 1.00 10.00 A

ATOM 996 OE1 GLN A 105 7.565 9.745 -3.728 1.00 10.00 A

ATOM 997 NE2 GLN A 105 9.040 10.667 -2.326 1.00 10.00 A

ATOM 998 HE21 GLN A 105 9.882 11.165 -2.243 1.00 10.00 A

ATOM 999 HE22 GLN A 105 8.499 10.388 -1.555 1.00 10.00 A

ATOM 1000 C GLN A 105 11.746 10.561 -6.552 1.00 10.00 A

ATOM 1001 O GLN A 105 12.181 11.702 -6.395 1.00 10.00 A

ATOM 1002 N LYS A 106 12.474 9.475 -6.319 1.00 10.00 A

ATOM 1003 HN LYS A 106 12.083 8.589 -6.484 1.00 10.00 A

ATOM 1004 CA LYS A 106 13.837 9.570 -5.812 1.00 10.00 A

ATOM 1005 CB LYS A 106 14.242 8.273 -5.098 1.00 10.00 A

ATOM 1006 CG LYS A 106 14.622 7.135 -6.032 1.00 10.00 A

ATOM 1007 CD LYS A 106 15.040 5.898 -5.256 1.00 10.00 A

ATOM 1008 CE LYS A 106 16.078 5.092 -6.021 1.00 10.00 A

ATOM 1009 NZ LYS A 106 17.407 5.759 -6.015 1.00 10.00 A

ATOM 1010 HZ1 LYS A 106 18.100 5.183 -6.531 1.00 10.00 A

ATOM 1011 HZ2 LYS A 106 17.738 5.881 -5.036 1.00 10.00 A

ATOM 1012 HZ3 LYS A 106 17.344 6.695 -6.465 1.00 10.00 A

ATOM 1013 C LYS A 106 14.844 9.913 -6.910 1.00 10.00 A

ATOM 1014 O LYS A 106 15.992 10.247 -6.620 1.00 10.00 A

ATOM 1015 N ALA A 107 14.411 9.841 -8.166 1.00 10.00 A

ATOM 1016 HN ALA A 107 13.480 9.592 -8.334 1.00 10.00 A

ATOM 1017 CA ALA A 107 15.287 10.131 -9.301 1.00 10.00 A

ATOM 1018 CB ALA A 107 14.749 9.489 -10.570 1.00 10.00 A

ATOM 1019 C ALA A 107 15.468 11.631 -9.502 1.00 10.00 A

ATOM 1020 O ALA A 107 15.141 12.174 -10.556 1.00 10.00 A

ATOM 1021 N GLN A 108 15.979 12.299 -8.484 1.00 10.00 A

ATOM 1022 HN GLN A 108 16.219 11.812 -7.664 1.00 10.00 A

ATOM 1023 CB GLN A 108 15.286 14.472 -7.580 1.00 10.00 A

ATOM 1024 CG GLN A 108 13.806 14.312 -7.885 1.00 10.00 A

ATOM 1025 CD GLN A 108 13.363 15.067 -9.126 1.00 10.00 A

ATOM 1026 OE1 GLN A 108 12.933 16.216 -9.044 1.00 10.00 A

ATOM 1027 NE2 GLN A 108 13.464 14.427 -10.280 1.00 10.00 A

ATOM 1028 HE21 GLN A 108 13.819 13.510 -10.275 1.00 10.00 A

ATOM 1029 HE22 GLN A 108 13.179 14.894 -11.095 1.00 10.00 A

ATOM 1030 CA GLN A 108 16.209 13.732 -8.548 1.00 10.00 A

ATOM 1031 C GLN A 108 17.660 14.033 -8.216 1.00 10.00 A

ATOM 1032 O GLN A 108 18.149 13.521 -7.187 1.00 10.00 A

ATOM 1033 OXT GLN A 108 18.309 14.767 -8.989 1.00 10.00 A

TER

ATOM 1034 CB LYS B 1 9.788 -21.126 1.556 1.00 10.00 B

ATOM 1035 CG LYS B 1 10.838 -22.219 1.651 1.00 10.00 B

ATOM 1036 CD LYS B 1 12.243 -21.649 1.522 1.00 10.00 B

ATOM 1037 CE LYS B 1 12.460 -21.015 0.156 1.00 10.00 B

ATOM 1038 NZ LYS B 1 13.846 -20.509 -0.017 1.00 10.00 B

ATOM 1039 HZ1 LYS B 1 14.123 -19.932 0.803 1.00 10.00 B

ATOM 1040 HZ2 LYS B 1 13.907 -19.923 -0.873 1.00 10.00 B

ATOM 1041 HZ3 LYS B 1 14.512 -21.304 -0.108 1.00 10.00 B

ATOM 1042 C LYS B 1 7.406 -20.414 1.775 1.00 10.00 B

ATOM 1043 O LYS B 1 6.262 -20.521 1.331 1.00 10.00 B

ATOM 1044 N LYS B 1 7.963 -22.575 0.741 1.00 10.00 B

ATOM 1045 HT1 LYS B 1 7.894 -22.099 -0.182 1.00 10.00 B

ATOM 1046 HT2 LYS B 1 7.041 -22.996 0.969 1.00 10.00 B

ATOM 1047 HT3 LYS B 1 8.668 -23.331 0.674 1.00 10.00 B

ATOM 1048 CA LYS B 1 8.352 -21.606 1.785 1.00 10.00 B

ATOM 1049 N SER B 2 7.881 -19.292 2.286 1.00 10.00 B

ATOM 1050 HN SER B 2 8.787 -19.284 2.660 1.00 10.00 B

ATOM 1051 CA SER B 2 7.100 -18.074 2.322 1.00 10.00 B

ATOM 1052 CB SER B 2 7.403 -17.325 3.618 1.00 10.00 B

ATOM 1053 OG SER B 2 7.695 -18.245 4.660 1.00 10.00 B

ATOM 1054 HG SER B 2 7.293 -19.100 4.451 1.00 10.00 B

ATOM 1055 C SER B 2 7.458 -17.212 1.116 1.00 10.00 B

ATOM 1056 O SER B 2 8.456 -17.476 0.434 1.00 10.00 B

ATOM 1057 N GLN B 3 6.650 -16.199 0.845 1.00 10.00 B

ATOM 1058 HN GLN B 3 5.867 -16.040 1.415 1.00 10.00 B

ATOM 1059 CA GLN B 3 6.901 -15.315 -0.282 1.00 10.00 B

ATOM 1060 CB GLN B 3 5.689 -14.435 -0.581 1.00 10.00 B

ATOM 1061 CG GLN B 3 4.793 -14.990 -1.678 1.00 10.00 B

ATOM 1062 CD GLN B 3 5.448 -14.952 -3.050 1.00 10.00 B

ATOM 1063 OE1 GLN B 3 6.672 -14.992 -3.175 1.00 10.00 B

ATOM 1064 NE2 GLN B 3 4.635 -14.892 -4.087 1.00 10.00 B

ATOM 1065 HE21 GLN B 3 3.658 -14.877 -3.912 1.00 10.00 B

ATOM 1066 HE22 GLN B 3 5.025 -14.856 -4.984 1.00 10.00 B

ATOM 1067 C GLN B 3 8.152 -14.470 -0.076 1.00 10.00 B

ATOM 1068 O GLN B 3 8.630 -14.301 1.049 1.00 10.00 B

ATOM 1069 N TRP B 4 8.680 -13.949 -1.171 1.00 10.00 B

ATOM 1070 HN TRP B 4 8.248 -14.114 -2.037 1.00 10.00 B

ATOM 1071 CA TRP B 4 9.877 -13.127 -1.120 1.00 10.00 B

ATOM 1072 CB TRP B 4 10.569 -13.065 -2.491 1.00 10.00 B

ATOM 1073 CG TRP B 4 9.729 -12.468 -3.583 1.00 10.00 B

ATOM 1074 CD1 TRP B 4 8.865 -13.127 -4.409 1.00 10.00 B

ATOM 1075 CD2 TRP B 4 9.684 -11.091 -3.976 1.00 10.00 B

ATOM 1076 NE1 TRP B 4 8.275 -12.245 -5.280 1.00 10.00 B

ATOM 1077 HE1 TRP B 4 7.611 -12.478 -5.969 1.00 10.00 B

ATOM 1078 CE2 TRP B 4 8.763 -10.990 -5.036 1.00 10.00 B

ATOM 1079 CE3 TRP B 4 10.328 -9.933 -3.528 1.00 10.00 B

ATOM 1080 CZ2 TRP B 4 8.474 -9.779 -5.659 1.00 10.00 B

ATOM 1081 CZ3 TRP B 4 10.039 -8.733 -4.147 1.00 10.00 B

ATOM 1082 CH2 TRP B 4 9.120 -8.665 -5.199 1.00 10.00 B

ATOM 1083 C TRP B 4 9.573 -11.725 -0.603 1.00 10.00 B

ATOM 1084 O TRP B 4 8.427 -11.267 -0.636 1.00 10.00 B

ATOM 1085 N ASN B 5 10.607 -11.065 -0.114 1.00 10.00 B

ATOM 1086 HN ASN B 5 11.486 -11.502 -0.098 1.00 10.00 B

ATOM 1087 CA ASN B 5 10.497 -9.710 0.405 1.00 10.00 B

ATOM 1088 CB ASN B 5 9.975 -9.723 1.844 1.00 10.00 B

ATOM 1089 CG ASN B 5 9.607 -8.339 2.345 1.00 10.00 B

ATOM 1090 OD1 ASN B 5 9.237 -7.459 1.567 1.00 10.00 B

ATOM 1091 ND2 ASN B 5 9.696 -8.140 3.652 1.00 10.00 B

ATOM 1092 HD21 ASN B 5 9.990 -8.886 4.218 1.00 10.00 B

ATOM 1093 HD22 ASN B 5 9.461 -7.256 4.002 1.00 10.00 B

ATOM 1094 C ASN B 5 11.870 -9.059 0.346 1.00 10.00 B

ATOM 1095 O ASN B 5 12.881 -9.719 0.596 1.00 10.00 B

ATOM 1096 N ASN B 6 11.912 -7.785 -0.006 1.00 10.00 B

ATOM 1097 HN ASN B 6 11.072 -7.307 -0.177 1.00 10.00 B

ATOM 1098 CA ASN B 6 13.176 -7.065 -0.112 1.00 10.00 B

ATOM 1099 CB ASN B 6 13.092 -5.876 -1.088 1.00 10.00 B

ATOM 1100 CG ASN B 6 11.778 -5.107 -1.032 1.00 10.00 B

ATOM 1101 OD1 ASN B 6 11.168 -4.947 0.024 1.00 10.00 B

ATOM 1102 ND2 ASN B 6 11.332 -4.620 -2.179 1.00 10.00 B

ATOM 1103 HD21 ASN B 6 11.863 -4.775 -2.991 1.00 10.00 B

ATOM 1104 HD22 ASN B 6 10.482 -4.135 -2.174 1.00 10.00 B

ATOM 1105 C ASN B 6 13.700 -6.629 1.250 1.00 10.00 B

ATOM 1106 O ASN B 6 12.963 -6.083 2.064 1.00 10.00 B

ATOM 1107 N ASP B 7 14.977 -6.887 1.490 1.00 10.00 B

ATOM 1108 HN ASP B 7 15.511 -7.332 0.799 1.00 10.00 B

ATOM 1109 CA ASP B 7 15.610 -6.517 2.749 1.00 10.00 B

ATOM 1110 CB ASP B 7 16.825 -7.404 3.034 1.00 10.00 B

ATOM 1111 CG ASP B 7 16.450 -8.796 3.507 1.00 10.00 B

ATOM 1112 OD1 ASP B 7 16.097 -8.951 4.698 1.00 10.00 B

ATOM 1113 OD2 ASP B 7 16.521 -9.744 2.691 1.00 10.00 B

ATOM 1114 C ASP B 7 16.036 -5.059 2.702 1.00 10.00 B

ATOM 1115 O ASP B 7 15.884 -4.318 3.676 1.00 10.00 B

ATOM 1116 N ASN B 8 16.566 -4.654 1.559 1.00 10.00 B

ATOM 1117 HN ASN B 8 16.656 -5.295 0.822 1.00 10.00 B

ATOM 1118 CA ASN B 8 17.006 -3.284 1.357 1.00 10.00 B

ATOM 1119 CB ASN B 8 18.436 -3.220 0.800 1.00 10.00 B

ATOM 1120 CG ASN B 8 18.840 -4.451 0.006 1.00 10.00 B

ATOM 1121 OD1 ASN B 8 18.549 -4.566 -1.187 1.00 10.00 B

ATOM 1122 ND2 ASN B 8 19.525 -5.374 0.661 1.00 10.00 B

ATOM 1123 HD21 ASN B 8 19.731 -5.216 1.611 1.00 10.00 B

ATOM 1124 HD22 ASN B 8 19.810 -6.178 0.174 1.00 10.00 B

ATOM 1125 C ASN B 8 16.042 -2.557 0.433 1.00 10.00 B

ATOM 1126 O ASN B 8 15.977 -2.845 -0.763 1.00 10.00 B

ATOM 1127 N PRO B 9 15.261 -1.623 0.988 1.00 10.00 B

ATOM 1128 CA PRO B 9 14.285 -0.856 0.226 1.00 10.00 B

ATOM 1129 CB PRO B 9 13.254 -0.496 1.293 1.00 10.00 B

ATOM 1130 CG PRO B 9 14.041 -0.368 2.555 1.00 10.00 B

ATOM 1131 CD PRO B 9 15.262 -1.243 2.411 1.00 10.00 B

ATOM 1132 C PRO B 9 14.881 0.412 -0.383 1.00 10.00 B

ATOM 1133 O PRO B 9 16.078 0.682 -0.254 1.00 10.00 B

ATOM 1134 N LEU B 10 14.035 1.188 -1.044 1.00 10.00 B

ATOM 1135 HN LEU B 10 13.094 0.918 -1.115 1.00 10.00 B

ATOM 1136 CA LEU B 10 14.468 2.426 -1.674 1.00 10.00 B

ATOM 1137 CB LEU B 10 13.605 2.737 -2.900 1.00 10.00 B

ATOM 1138 CG LEU B 10 13.977 2.018 -4.203 1.00 10.00 B

ATOM 1139 CD1 LEU B 10 13.758 0.516 -4.091 1.00 10.00 B

ATOM 1140 CD2 LEU B 10 13.188 2.590 -5.370 1.00 10.00 B

ATOM 1141 C LEU B 10 14.425 3.582 -0.684 1.00 10.00 B

ATOM 1142 O LEU B 10 15.177 4.547 -0.806 1.00 10.00 B

ATOM 1143 N PHE B 11 13.545 3.470 0.300 1.00 10.00 B

ATOM 1144 HN PHE B 11 12.986 2.670 0.349 1.00 10.00 B

ATOM 1145 CA PHE B 11 13.397 4.491 1.326 1.00 10.00 B

ATOM 1146 CB PHE B 11 12.036 5.184 1.212 1.00 10.00 B

ATOM 1147 CG PHE B 11 11.948 6.198 0.105 1.00 10.00 B

ATOM 1148 CD1 PHE B 11 10.855 6.216 -0.747 1.00 10.00 B

ATOM 1149 CD2 PHE B 11 12.950 7.138 -0.080 1.00 10.00 B

ATOM 1150 CE1 PHE B 11 10.764 7.150 -1.761 1.00 10.00 B

ATOM 1151 CE2 PHE B 11 12.866 8.074 -1.094 1.00 10.00 B

ATOM 1152 CZ PHE B 11 11.771 8.080 -1.935 1.00 10.00 B

ATOM 1153 C PHE B 11 13.553 3.865 2.707 1.00 10.00 B

ATOM 1154 O PHE B 11 13.127 2.730 2.934 1.00 10.00 B

ATOM 1155 N LYS B 12 14.176 4.591 3.622 1.00 10.00 B

ATOM 1156 HN LYS B 12 14.494 5.491 3.388 1.00 10.00 B

ATOM 1157 CA LYS B 12 14.382 4.090 4.971 1.00 10.00 B

ATOM 1158 CB LYS B 12 15.869 3.862 5.254 1.00 10.00 B

ATOM 1159 CG LYS B 12 16.447 2.619 4.592 1.00 10.00 B

ATOM 1160 CD LYS B 12 17.787 2.238 5.205 1.00 10.00 B

ATOM 1161 CE LYS B 12 18.233 0.856 4.749 1.00 10.00 B

ATOM 1162 NZ LYS B 12 19.327 0.318 5.598 1.00 10.00 B

ATOM 1163 HZ1 LYS B 12 19.544 -0.664 5.331 1.00 10.00 B

ATOM 1164 HZ2 LYS B 12 19.043 0.335 6.597 1.00 10.00 B

ATOM 1165 HZ3 LYS B 12 20.184 0.893 5.484 1.00 10.00 B

ATOM 1166 C LYS B 12 13.791 5.034 6.008 1.00 10.00 B

ATOM 1167 O LYS B 12 14.001 6.245 5.952 1.00 10.00 B

ATOM 1168 N SEP B 13 13.046 4.473 6.948 1.00 10.00 B

ATOM 1169 HN SEP B 13 12.898 3.507 6.926 1.00 10.00 B

ATOM 1170 CA SEP B 13 12.433 5.256 8.006 1.00 10.00 B

ATOM 1171 CB SEP B 13 11.167 4.560 8.503 1.00 10.00 B

ATOM 1172 OG SEP B 13 10.346 5.443 9.246 1.00 10.00 B

ATOM 1173 P SEP B 13 9.490 4.877 10.469 1.00 10.00 B

ATOM 1174 O1P SEP B 13 10.284 4.181 11.494 1.00 10.00 B

ATOM 1175 O2P SEP B 13 8.518 5.833 11.026 1.00 10.00 B

ATOM 1176 O3P SEP B 13 8.592 3.726 9.834 1.00 10.00 B

ATOM 1177 C SEP B 13 13.425 5.463 9.153 1.00 10.00 B

ATOM 1178 O SEP B 13 13.312 4.855 10.220 1.00 10.00 B

ATOM 1179 N ALA B 14 14.418 6.307 8.910 1.00 10.00 B

ATOM 1180 HN ALA B 14 14.461 6.754 8.035 1.00 10.00 B

ATOM 1181 CA ALA B 14 15.440 6.602 9.898 1.00 10.00 B

ATOM 1182 CB ALA B 14 16.453 5.467 9.956 1.00 10.00 B

ATOM 1183 C ALA B 14 16.137 7.908 9.544 1.00 10.00 B

ATOM 1184 O ALA B 14 15.962 8.432 8.443 1.00 10.00 B

ATOM 1185 N THR B 15 16.916 8.431 10.473 1.00 10.00 B

ATOM 1186 HN THR B 15 17.010 7.973 11.334 1.00 10.00 B

ATOM 1187 CA THR B 15 17.639 9.670 10.249 1.00 10.00 B

ATOM 1188 CB THR B 15 17.758 10.485 11.549 1.00 10.00 B

ATOM 1189 OG1 THR B 15 18.037 9.600 12.644 1.00 10.00 B

ATOM 1190 HG1 THR B 15 18.994 9.570 12.787 1.00 10.00 B

ATOM 1191 CG2 THR B 15 16.465 11.234 11.824 1.00 10.00 B

ATOM 1192 C THR B 15 19.029 9.387 9.685 1.00 10.00 B

ATOM 1193 O THR B 15 19.993 9.214 10.431 1.00 10.00 B

END
